# Characterization of the *Cannabis sativa* glandular trichome epigenome

**DOI:** 10.1186/s12870-024-05787-x

**Published:** 2024-11-14

**Authors:** Lee J. Conneely, Bhavna Hurgobin, Sophia Ng, Muluneh Tamiru-Oli, Mathew G. Lewsey

**Affiliations:** 1 La Trobe Institute for Sustainable Agriculture and Food, La Trobe University, AgriBio Building, Bundoora, VIC 3086 Australia; 2 Australian Research Council Research Hub for Medicinal Agriculture, La Trobe University, AgriBio Building, Bundoora, VIC 3086 Australia; 3 Australian Research Council Centre of Excellence in Plants for Space, La Trobe University, Bundoora, VIC Australia

**Keywords:** *Cannabis sativa*, Glandular trichomes, Specialised metabolism, Multiomics, H3K4me3, H3K27me3, H3K56ac, H2A.Z, Chromatin immunoprecipitation, Gene regulation, Cis-regulatory element

## Abstract

**Background:**

The relationship between epigenomics and plant specialised metabolism remains largely unexplored despite the fundamental importance of epigenomics in gene regulation and, potentially, yield of products of plant specialised metabolic pathways. The glandular trichomes of *Cannabis sativa* are an emerging model system that produce large quantities of cannabinoid and terpenoid specialised metabolites with known medicinal and commercial value. To address this lack of epigenomic data, we mapped H3K4 trimethylation, H3K56 acetylation, H3K27 trimethylation post-translational modifications and the histone variant H2A.Z, using chromatin immunoprecipitation, in *C. sativa* glandular trichomes, leaf, and stem tissues. Corresponding transcriptomic (RNA-seq) datasets were integrated, and tissue-specific analyses conducted to relate chromatin states to glandular trichome specific gene expression.

**Results:**

The promoters of cannabinoid and terpenoid biosynthetic genes, specialised metabolite transporter genes, defence related genes, and starch and sucrose metabolism were enriched specifically in trichomes for histone marks H3K4me3 and H3K56ac, consistent with active transcription. We identified putative trichome-specific enhancer elements by identifying intergenic regions of H3K56ac enrichment, a histone mark that maintains enhancer accessibility, then associated these to putative target genes using the tissue specific gene transcriptomic data. Bi-valent chromatin loci specific to glandular trichomes, marked with H3K4 trimethylation and H3K27 trimethylation, were associated with genes of MAPK signalling pathways and plant specialised metabolism pathways, supporting recent hypotheses that implicate bi-valent chromatin in plant defence. The histone variant H2A.Z was largely found in intergenic regions and enriched in chromatin that contained genes involved in DNA homeostasis.

**Conclusion:**

We report the first genome-wide histone post-translational modification maps for *C. sativa* glandular trichomes, and more broadly for glandular trichomes in plants. Our findings have implications in plant adaptation and stress responses and provide a basis for enhancer-mediated, targeted, gene transformation studies in plant glandular trichomes.

**Supplementary Information:**

The online version contains supplementary material available at 10.1186/s12870-024-05787-x.

## Background

Histone post-translational modifications and histone variants are features of plant epigenomes that influence gene regulation and, through this, tissue and cell functionality [1–3]. H3K4 trimethylation (H3K4me3) and H3K27 trimethylation (H3K27me3) are the most extensively studied histone post-translational modifications, and have opposing functions on gene expression [4–8]. H3K4me3 is deposited by trithorax complexes in the 5’ untranslated region (UTR) of actively transcribed genes where it is required for RNA polymerase II pause-release and transcriptional elongation [9–11]. Conversely, H3K27me3 is deposited by polycomb repressor complex 2 (PRC2) and contributes to the formation of facultative heterochromatin, referred to as polycomb chromatin, that is enriched in the gene body of repressed genes. Furthermore, H3K27me3 PRC2 mediated gene repression operates synergistically with polycomb repressor complex 1 (PRC1) in the monoubiquitylation of the histone variant H2A.Z to repress gene expression [12, 13]. Similar to H3K27me3, H2A.Z in plants is enriched in the gene body of repressed genes [14].

Despite their antagonistic effects on gene expression, H3K4me3 and H3K27me3 may also co-localise on the same nucleosome in a phenomenon known as bivalency. It is hypothesised that bi-valent chromatin containing antagonistic histone post-translational modifications results in poised states of gene expression, whereby plants can rapidly upregulate and fine tune spatio-temporal gene expression in response to various environmental cues [15]. Vernalization is a well-studied example of H3K4me3-H3K27me3 bivalency in plants, whereby temperatures influence the ratio of H3K4me3 to H3K27me3 at a bi-valent locus associated with the floral development repressor gene *FLC* [16]. This modulates *FLC* expression through gene silencing, allowing floral development when temperature increases in spring.

Enhancer elements are short stretches of DNA that contain transcription factor binding motifs of approximately 7–22 bp, found in both intergenic and intragenic regions [17, 18]. Interactions between enhancer elements and gene promoters drive gene transcription, and individual enhancers can regulate single or multiple target genes [19]. Intergenic enhancers may regulate expression of proximal (≤ 1.5 Kbp) genes, or distal genes that are located from thousands to a million base pairs away (termed distal enhancer elements) [20–22]. Distal enhancer elements mediate gene expression via long-range chromatin interactions known as chromatin looping [22, 23]. Enhancers can drive tissue and cell-type specific transcription, making them a useful resource to understand patterns of gene regulation and a useful tool in biotechnology where precise expression in certain cells may be required [24, 25].

The chromatin features specific to enhancers can be used to discover novel regulatory elements [26–28]. For example, plant distal enhancer elements are typically flanked by narrow regions of H3K56 acetylation (H3K56ac) in the tissue or cell type where they are active, a mark that promotes a euchromatic environment and permits transcription factor binding [29, 30]. H3K56ac is also enriched in the promoters of transcribed genes, similar to H3K4me3, where it promotes gene expression [29]. By mapping and analysing the genome wide distribution of H3K56ac, then cross-referencing it with the distribution of genes and intergenic space, undiscovered putative distal enhancer elements can be identified.

*Cannabis sativa* is a predominantly dioecious, dicotyledonous, plant of the Cannabaceae family that has been cultivated for at least 2700 years for both its long durable fibres and its medicinal properties [31–33]. *C. sativa* is known for the production of cannabinoids, a group of species-specific terpenophenolic compounds noted with medicinal and psychoactive properties [34, 35]. The organs that produce specialised metabolites like cannabinoids, the glandular trichomes, have garnered the interest of the plant science community as potential new models for the study of plant cell and tissue development [36]. Most recently there has been interest in engineering of glandular trichomes, due to the remarkable quantities of specialised metabolite they are able to produce and sequester away from other plant tissues (e.g. THC 14.98 +/- 2.23% dry weight) [37–40]. However, for glandular trichome engineering to be feasible in *C. sativa* we must understand and be able to manipulate trichome-specific gene expression and specialised metabolism. This would be enabled by mapping epigenomic features, such as histone modifications and variants, and relating them to gene expression characteristics. There is currently a lack of any such data in *C. sativa* or glandular trichomes more broadly.

In this study we generated genome-wide maps of histone post translational modifications H3K4me3, H3K27me3, H3K56ac, and the histone variant H2A.Z for *C. sativa* glandular trichomes, leaves and stems. We made comparisons between tissues to identify trichome specific epigenomic signals. Matched transcriptomic (RNA-seq) datasets were generated for each tissue type to validate histone function in relation to gene expression and to enable functions associated with different epigenome features to be analysed. The relationships of these functions with plant specialised metabolism, abiotic and biotic stress resistance were assessed. Lastly, putative glandular trichome specific distal enhancer elements were predicted by examining H3K56ac data across tissues and drawing comparisons to differential gene expression analysis.

## Results

### Mapping histone marks in three *C. sativa* tissues by chromatin immunoprecipitation sequencing

**Fig. 1:**
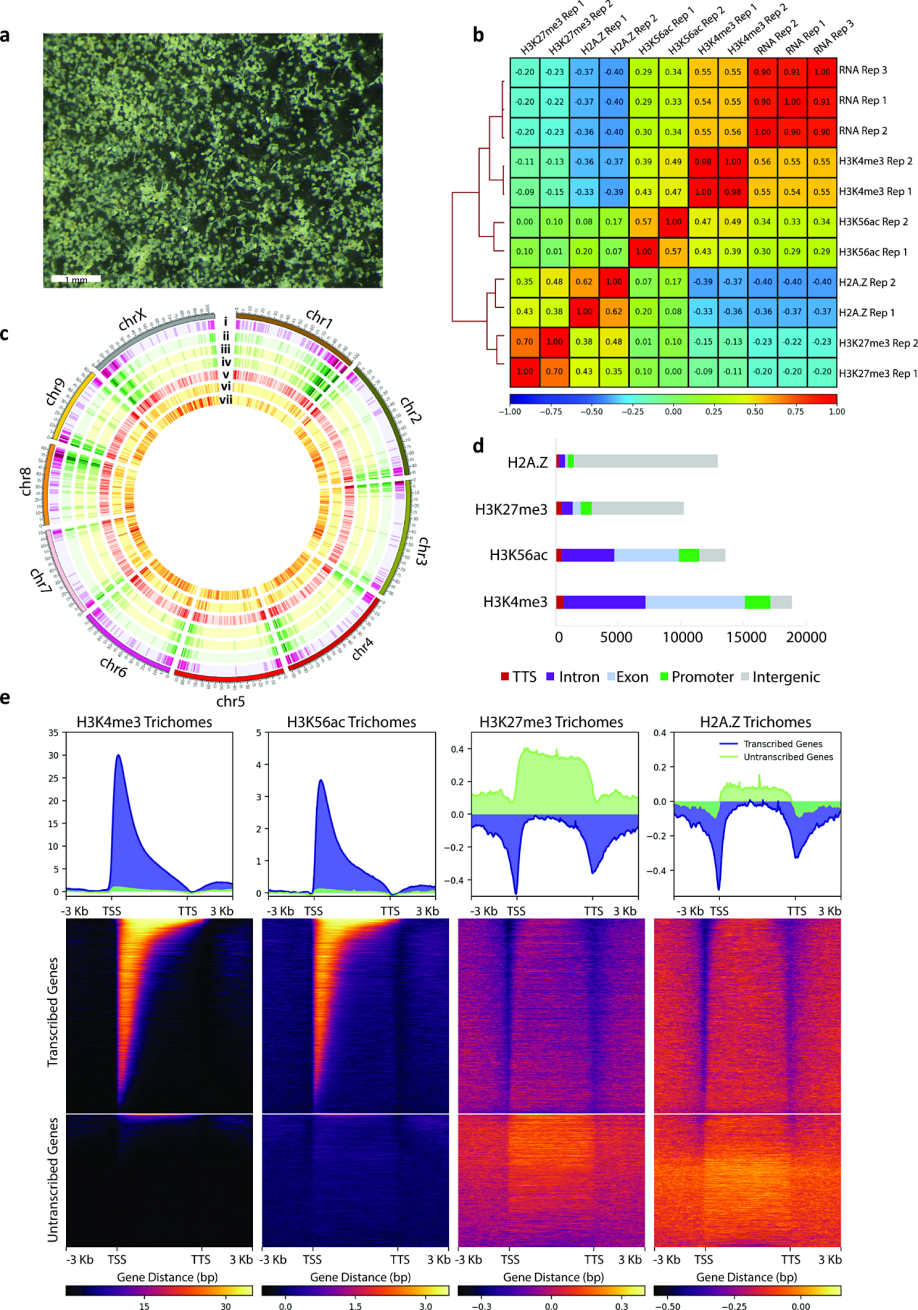
Assessment of data quality and genome feature associations of histone marks in *Cannabis sativa* glandular trichomes. (**a**) Light microscopy image (1 mm scale bar) of *C. sativa* glandular trichomes collected using a 25 μm mesh size during floral tissue ice-water extraction method. (**b**) Spearman correlation matrix of bam files for 3 biological replicates of glandular trichome RNA-seq data and two biological replicates each of glandular trichome ChIP-seq data for H3K4me3, H3K56ac, H3K27me3, and H2A.Z. (**c**) Distribution of features across the *C. sativa* genome; (**i**) gene density plot (**ii**) glandular trichome transcribed-gene density plot (**iii**) glandular trichome H3K4me3 peak density plot (iv) glandular trichome H3K56ac peak density plot (**v**) glandular trichome untranscribed-gene density plot (**vi**) glandular trichome H3K27me3 peak density plot and (**vii**) glandular trichome H2A.Z density plot. (**d**) Distribution of H2A.Z, H3K27me3, H3K56ac, and H3K4me3 across genome features. (**e**) Distribution of H3K4me3, H3K56ac, H3K27me3, and H2A.Z reads across all transcribed genes and untranscribed genes in glandular trichomes. Scale bars indicate relative enrichment

We first generated genome wide maps of histone post-translational modifications H3K56ac, H3K4me3, H3K27me3, and histone variant H2A.Z, in *C. sativa* tissues. A chromatin immunoprecipitation sequencing (ChIP-seq) protocol was first optimised for cannabis tissues, to avoid interference with procedures by the extreme concentrations of specialized metabolites (Additional File 1: Fig S1). The protocol was then applied to glandular trichome (Fig. 1a), stem (internode), and vegetative leaf tissues and sequencing libraries generated (Additional File 1: Fig. S2, Table S1, Table S2). Alignment of the sequencing data to the *C. sativa* reference genome was successful (Additional File 1: Table S3a). Quality control fingerprint plots were consistent with the expected distributions for narrow peak H3K4me3 and H3K56ac and broad peak H3K27me3 and H2A.Z data, respectively (Additional File 1; Fig. S3). Corresponding RNA-seq libraries were generated and analysed in triplicate for integration with the ChIP-seq data (Additional File 1: Fig. S4, Table S3b).

### Associations of histone marks with gene expression in *C. sativa*

Characteristic association between histone marks and gene expression have been observed across many plant species [41]. We examined if these associations were conserved in *C. sativa* (Fig. 1b-c, Additional File 1: Fig. S5). The distribution of RNA-seq reads – indicative of gene expression - positively correlated with the distributions of ChIP-Seq reads for H3K56ac, H3K4me3 in each tissue (Spearman correlation coefficient, Fig. 1b). Contrastingly, H3K27me3 and H2A.Z ChIP-Seq reads tended to negatively correlate with RNA-seq reads. Consistent with these results, actively transcribed genes were observed in a genome wide visualization overlaying reads for both H3K4me3 and H3K56ac, whilst un-transcribed genes overlayed H3K27me3 and H2A.Z (Fig. 1c). The majority of H3K27me3 and H2A.Z ChIP-seq reads mapped to intergenic regions, whereas the majority of H3K4me3 and H3K56ac reads mapped to gene features (Fig. 1d). H3K4me3 and H3K56ac were enriched at the transcriptional start site of actively transcribed genes, consistent with their roles in facilitating transcription [9, 42, 43]. Conversely, H3K27me3 and H2A.Z were enriched in the gene bodies of un-transcribed genes, consistent with their synergistic roles in polycomb mediated gene repression [14, 44] (Fig. 1e, Additional File 1: Fig. S6). Overall, the distribution of histone marks was consistent with observations in other species [30, 43].

### Histone marks co-localise in the *C. sativa* genome which may reflect their influence on gene activation or repression

Histone marks can function co-operatively to either promote or repress the expression of genes. H3K56ac often co-localises with H3K4me3 at transcriptional start sites (TSS) and promoters to promote a relaxed chromatin environment conducive for gene expression, while H3K27me3 and mono ubiquitination of H2A.Z co-localise in the body of repressed genes to promote polycomb mediated gene silencing [12, 44]. We investigated the extent of these functional co-occurrences in *C. sativa.* To do so we identified high-confidence loci, genome-wide, that were enriched for each individual mark.

**Fig. 2:**
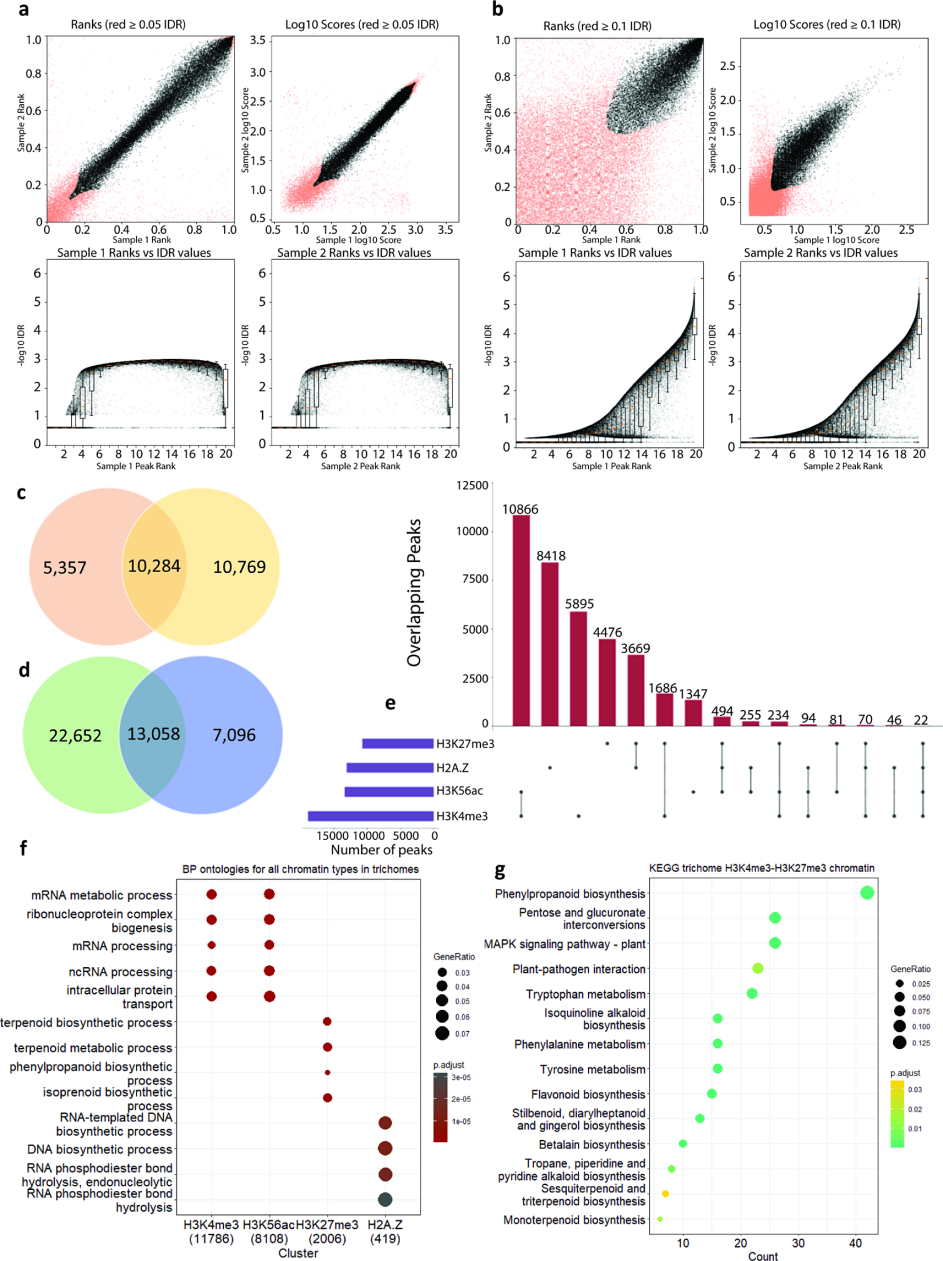
Identification of high confidence, replicable, histone mark loci, and their general functional associations in glandular trichomes. Replicable peaks (black points) for (**a**) H3K4me3 narrow peaks and (**b**) H3K56ac mixed peak type in glandular trichomes were determined by applying irreproducible discovery rate (IDR) analysis, comparing peak ranks by significance value between sample panels show good replicability plotted by both sample peak ranks (top panels) and corresponding -log10 q-value of peak enrichment vs. sample rank (bottom panels) indicates the strength of enrichment (-log10 q-value) across ranked list. H3K4me3 showed extremely high replicability with the majority of peaks displaying strong enrichment across lower ranks (bottom panel) yielding 19,704 replicable peaks, and H3K56ac and showed good replicability yielding 13,590 replicable peaks. Replicable broad peaks determined via the intersection of replicates for (**c**) H3K27me3 and (**d**) H2A.Z yield 10,284 and 13,058 replicable peaks respectively for glandular trichomes. (**e**) Upset plot of the replicable peak type data showing the relationships, by means of physical intersection of the various chromatin types throughout the glandular trichome epigenome. (**f**) Biological process ontologies enriched in glandular trichomes for each H3K4me3, H3K56ac, H3K27me3, and H2A.Z. (**g**) KEGG ontology analysis of supposed bivalent chromatin H3K4me3-H3K27me3 in glandular trichomes

Different strategies were necessary for H3K4me3 and H3K56Kac, because these are found across smaller loci called “narrow” or “mixed” peaks in ChIP-Seq analyses, relative to H3K27me3 and H2A.Z, which are found across large loci termed “broad” peaks. Accordingly, we applied defined gold standard community practises [45]. The Irreproducible Discovery Rate (IDR) was applied to H3K4me3 and H3K56Kac data to ensure the statistical replicability of peak locations across our two biologically independent replicates (IDR cutoff ≤ 0.05 for H3K4me3 due to the true narrow peaks, and a less stringent IDR ≤ 0.10 for H3K56ac due to the mixed peak-type). Replicate peak ranks indicated a high degree of replicability (black points) for H3K4me3 data, and acceptable replicability in H3K56ac data (Fig. 2a-b). This resulted in 19,704 (H3K4me3) and 13,590 (H3K56Kac) high confidence peaks for glandular trichomes, 18,668 (H3K4me3) and 14,147 (H3K56ac) high confidence peaks for stem (internode), and 15,135 (H3K4me3) and 1,655 (H3K56ac) high confidence peaks for leaf tissue (Fig. 2a-b, Additional File 1: Fig. S7). Broad domain peak replicability was ensured by retaining only peaks that were found in both biological replicates, yielding 10,284 and 13,058 high confidence peaks for H3K27me3 and H2A.Z in *C. sativa* glandular trichomes respectively (Fig. 2c-d). The genomic locations of high-confidence peaks and their associated features were then annotated for all histone marks (Additional Files 2–4). Sequence reads and high confidence peaks for each histone mark were also loaded into a public web browser (https://jbrowse.latrobe.edu.au/cannabis_trichome_epigenome/) to allow individual loci to be examined.

We then investigated potential co-localisation in the genome of histone marks, focusing on glandular trichomes (Fig. 2e). The co-localization of H3K4me3 and H3K56ac was statistically significant (Fisher exact test *p* < 1 × 10^− 5^; 10,866 overlapping peaks *versus* 5,895 H3K4me3 and 1,347 H3K56ac non-overlapping peaks) (Additional File 1: Fig. S7b-d). The co-localisation of H3K27me3 and H2A.Z was also statistically significant (Fisher exact test *p* < 1 × 10^− 5^; 3,669 overlapping peaks), as was co-localisation of H3K4me3 and H3K27me3 (Fisher exact test *p* < 1 × 10^− 5^; 1,686 overlapping peaks). We further cross-referenced replicable peaks for each histone mark against our trichome RNA-seq dataset – we partitioned genes according to their transcriptional status (transcribed genes ≥ 1 TPM) and then recorded genes that contained each of the four histone marks (Additional File 1: Table S4).

### Cross-validation of the expression-associated histone mark H3K4me3 across independent public transcriptome datasets

We made use of public data to cross-validate our ChIP-seq maps. Several laboratories have performed transcriptomic analyses of *C. sativa* trichomes [46–48]. Some variation between datasets would be expected for both technical and biological reasons, such as different *C. sativa* strains, varying trichome purification methods, differences in sequence depth and sequencing design such as single and paired end sequencing methods. Nonetheless, we can reasonably expect that the population of expressed genes should substantially correlate between trichome datasets, because the fundamental biological processes operating in glandular trichomes of any *C. sativa* strain will not differ. It has been confidently established in many plant species that H3K4me3 is directly associated with the TSSs of expressed genes [5, 9, 49, 50]. This consequently provided an opportunity to cross-validate the accuracy of our ChIP-seq maps across the independent transcriptomic datasets. We extracted the population of genes underlying glandular trichome specific H3K4me3 loci in our ChIP-seq dataset, then quantified their expression in each of 33 transcriptome datasets (Additional File 1: Fig. S7e). We observed positive correlation of the expression of this population of genes across all trichome specific transcriptome datasets, contrasting with lower correlation to *C. sativa* stem and leaf specific transcriptomes. This indicates that our ChIP-seq method robustly identifies genes expressed trichome-specifically, consistent with the known biology of H3K4me3.

### Histone marks are associated with different functional categories of genes in glandular trichomes

Next, we asked what the function of genes associated with different histone marks in glandular trichomes were. To gain a global understanding, we assessed all loci bearing histone marks in glandular trichomes; we did not focus on loci only present specifically in glandular trichomes. Gene Ontology (GO) enrichment analysis of biological processes (BP) was performed on genes found within regions bearing each of the four histone marks in glandular trichomes (Fig. 2f). The genes associated with H3K4me3 (11,786 genes) and H3K56ac (8,108 genes) were primarily enriched for the same housekeeping functions, including mRNA metabolic process, and mRNA processing. This likely reflects the conserved role of H3K4me3 and H3K56ac in promoting gene expression, and the fact that our analyses considered all expressed genes rather than a trichome-specific subset. H3K27me3 and H2A.Z loci contained fewer genes and 419, respectively). Genes associated H3K27me3 (2,006 genes) were enriched for functions related to plant specialised metabolism, such as terpenoid metabolic process and isoprenoid biosynthetic process. This was notable, as H3K27me3 is associated with repression of gene expression. Genes associated with H2A.Z (419 genes) were enriched for functions associated with DNA homoeostasis and transcriptional control, in line with previous observations made in plants and animals (Additional File 5) [51, 52].

Our observations that specialised metabolism genes were enriched within H3K27me3 regions, and that H3K27me3 was frequently co-located with H3K4me3 in glandular trichomes, prompted us to investigate whether bi-valent chromatin states may be a common feature of regulation of specialised metabolism in glandular trichomes (Fig. 2e). Bi-valent H3K27me3/H3K18ac loci are involved in regulation of biosynthesis of the specialised metabolite camalexin in *A. thaliana* [53]. We examined this by using the Kyoto Encyclopedia for Genes and Genomes (KEGG) curated pathways for *C. sativa* cs10 (GCF_900626175.2) to analyse pathways enriched amongst genes located in regions where H3K27me3/H3K4me3 overlapped. Pathways enriched amongst these genes were associated with plant specialised metabolism and defence, including phenylpropanoid biosynthesis, MAPK signalling, plant-pathogen interaction, flavonoid biosynthesis, and sesquiterpenoid biosynthesis (Fig. 2g, Additional File 6).

### Differential epigenomic analysis shows glandular trichome specific enrichment of histone marks associated with specialised metabolism

**Fig. 3:**
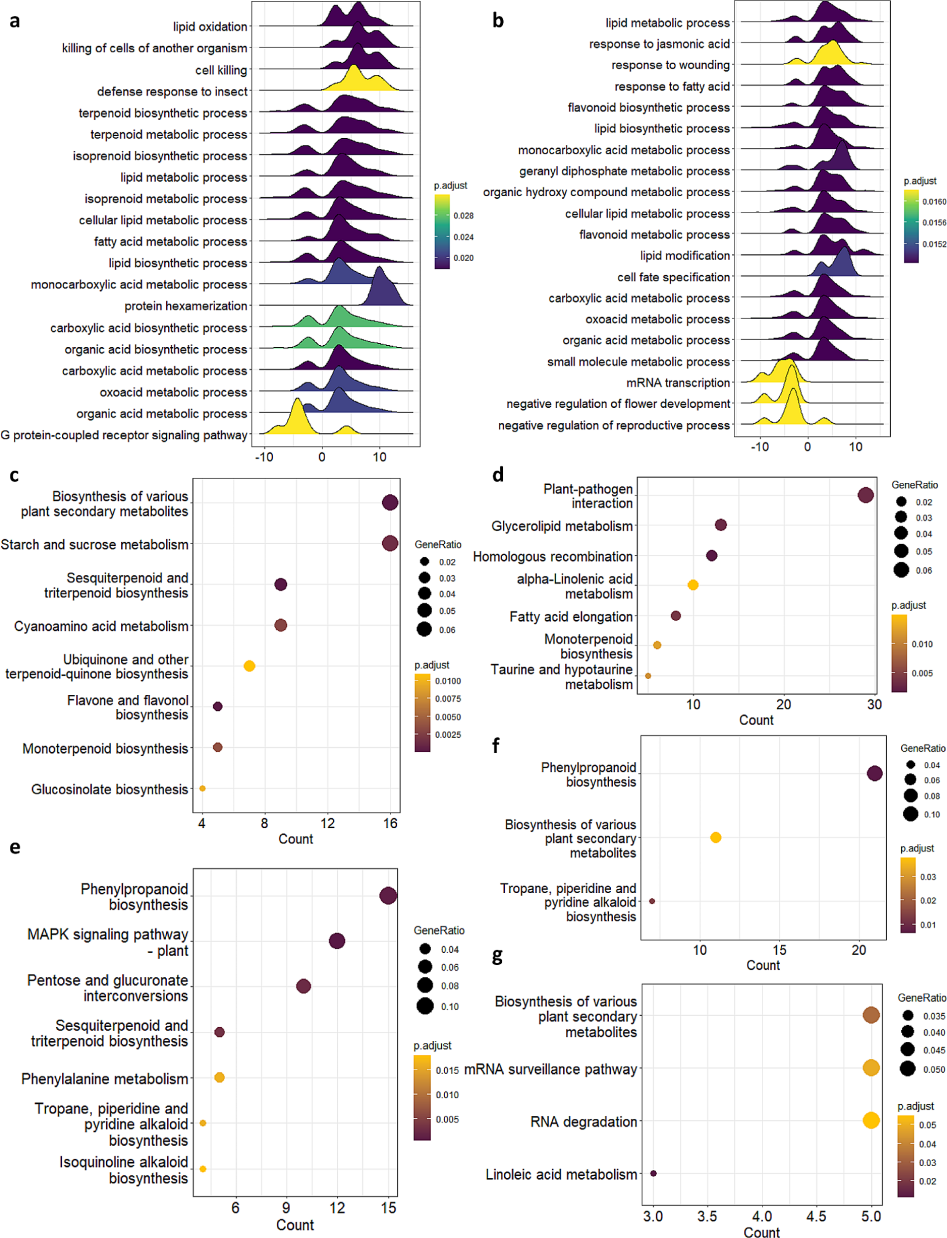
Analysis of glandular trichome specific gene expression and histone marks yields functional insights. (**a**) Pairwise gene set enrichment analysis (GSE) leveraging Leaf and (**b**) Stem RNA datasets. KEGG ontology analysis of glandular trichome specific chromatin types (**c**) H3K4me3 (**d**) H3K56ac (**e**) H3K4me3-H3K27me3 (**f**) H3K27me3 and (**g**) H2A.Z

We reasoned that by focusing our analysis on glandular trichome specific gene expression and histone marks, we could gain deeper insight into gene regulation in glandular trichomes. To achieve this we first identified differentially expressed genes in glandular trichomes versus leaf or stem tissues. There were 1,765 (trichome vs. leaf) and 1,987 (trichome vs. stem) differentially expressed genes (log 2FC > 2, BH p.adj < 0.05) (Additional File 7). Next, we assessed the functions of genes differentially expressed between these tissues using gene set enrichment analysis (GSE). Genes differentially expressed in glandular trichomes, compared with both stem and leaf, were highly enriched for biological processes including lipid metabolism, specialised metabolism, and organic acid biosynthesis in both cases (Fig. 3a-b, Additional File 8). These genes were also enriched for molecular functions including delta-9 tetrahydrocannabinolate synthase activity, cannabidiolate synthase activity, germacrene-a synthase activity, and other specialised metabolite related functions (Additional File 1: Fig. S8a-b, Additional File 8).

We next examined the functions of genes that were associated with glandular trichome specific regions of the different histone marks. To accomplish this, we identified the glandular trichome specific enriched loci for each histone mark from our high confidence peak lists (i.e. loci not present in any other tissue), then identified genes underlying these peaks. Functions enriched amongst these genes were assessed by KEGG ontology analysis (Additional File 9). Genes within glandular trichome specific H3K4me3 or H3K56ac regions were enriched for starch and sucrose metabolism, sesquiterpenoid and triterpenoid biosynthesis, plant-pathogen interactions, and monoterpenoid biosynthesis (Fig. 3c-d). Genes within glandular trichome specific H3K4me3-H3K27me3 bi-valent regions were enriched for MAPK signalling, phenylpropanoid biosynthesis, and sesquiterpenoid and triterpenoid biosynthesis (Fig. 3e). Genes within glandular trichome specific H3K27me3 chromatin or H2A.Z chromatin were enriched in KEGG ontologies related to biosynthesis of various plant specialised metabolites, phenylpropanoid biosynthesis, and mRNA surveillance (Fig. 3f-g). This analysis implies that specialised metabolism may be differentially regulated by specific histone marks in glandular trichomes (Additional File 9).

We then examined whether there were tissue specific differences in the histone marks associated with well characterised genes expressed specifically in glandular trichomes. For example, phytocannabinoid biosynthesis 2-acylphloroglucinol 4-prenyltransferase (also called cannabigerolic acid synthase, CBGAS/PT4; LOC115713185), ABC-transporter B family member 2 (LOC115716265), and alpha-humulene synthase (LOC115724563) were all expressed specifically in glandular trichomes and associated with glandular trichome specific enrichment of the transcription-associated histone marks H3K4me3 and H3K56ac in their promoter regions (Additional File 1: Fig. S9a) [9, 54]. The silencing marks H3K27me3 and H2A.Z were found in the gene body of alpha humulene synthase (LOC115724563) in stem and leaf tissues, consistent with the lack of expression of this gene in those tissues. H3K27me3 enrichment was less at alpha humulene synthase (LOC115724563) in glandular trichomes, where the gene is expressed, but it was not completely absent. Phytocannabinoid biosynthesis 2-acylphloroglucinol 4-prenyltransferase (LOC115713185) and ABC-transporter B family member 2 (LOC115716265) were not associated with H3K27me3 or H2A.Z in stem or leaf, despite their lack of expression in those tissues. Taken together, these observations indicate that genes expressed trichome specifically are associated with trichome specific local chromatin landscapes but may also indicate further complexity or multivalency to chromatin states and the regulation of expression at individual genes in glandular trichomes (Additional File 1: Fig. S9a).

### Glandular trichome specific epigenomic regulation of putative gene clusters in *Cannabis sativa*

Genes encoding components of specialized metabolite pathways are frequently found in plant gene clusters (GCs) [55]. These can be the consequence of local duplications such as gene family expansion in terpene synthases or, alternatively, unrelated genes that constitute or modulate a metabolic pathway may be co-located thereby forming a biosynthetic gene cluster (BGC) [56–61]. Genes in either cluster type are frequently expressed tissue or cell type specifically [62, 63]. The epigenomic properties of such clusters are poorly defined in plants and understanding them better may offer a unique and refined strategy to introduce entire biosynthetic gene pathways, discretely into a genome, in a tissue or cell type specific manner for applications in plant metabolic engineering.

**Fig. 4:**
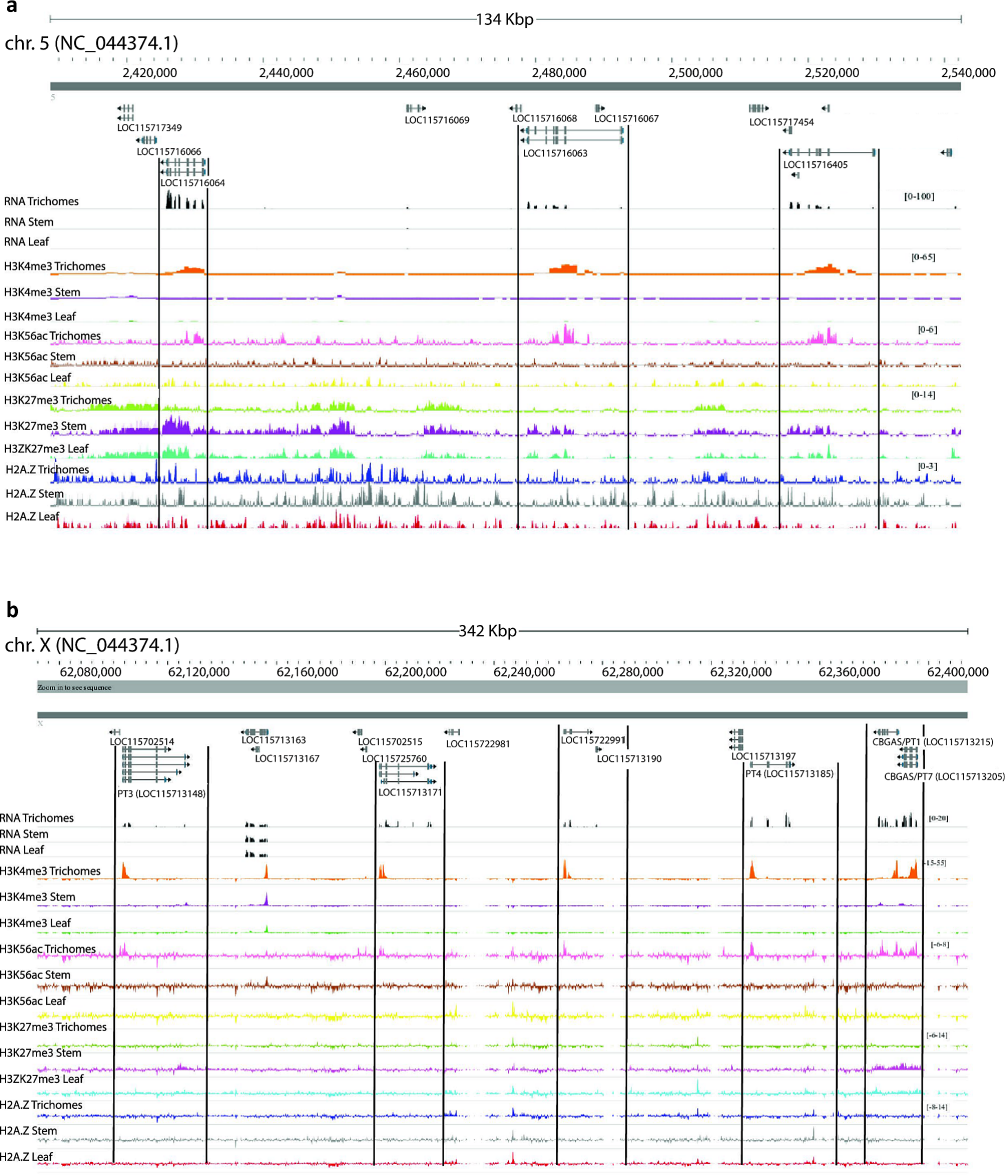
Specialised metabolism gene clusters have glandular trichome histone marks and gene expression. (**a**) Monoterpene synthase gene cluster (cluster no. 17) shows glandular trichome specific enrichment of H3K4me3 and H3K56ac chromatin and corresponding gene expression in LOC115716064 limonene synthase, LOC115716063 myrcene synthase, and LOC115716405 myrcene synthase and inverse depletion and expression in leaf and stem datasets with corresponding H3K27me3 enrichment in stem and leaf datasets. (**b**) A 342 Kbp region of X chromosome containing putative novel cannabigerolic acid synthase/ prenyltransferase gene cluster. This had histone marks H3K4me3 and H3K56ac specifically in glandular trichomes, conducive to gene expression, and corresponding trichome specific expression of PT3 (LOC115713148), LOC115713171, LOC115722991, PT4 (LOC115713185), CBGAS/PT1 (LOC115713215), and CBGAS/PT7 (LOC115713205)

We consequently examined the expression status of genes in putative *C. sativa* gene clusters and their associated histone marks, focusing upon two previously predicted clusters that may have glandular trichome specific activity [64]. The first putative BGC (#17), involved in monoterpene biosynthesis, exhibited glandular trichome specific gene expression, and association with transcriptionally active histone marks (H3K4me3 and H3K56ac), including limonene synthase/TPS1 (LOC115716064), and two myrcene synthases (LOC115716063, LOC115716405) (Fig. 4a). Contrastingly, in leaf and stem tissue these same genes were associated with the repressive mark H3K27me3. The second putative BGC (#28) had trichome specific gene expression of class V chitinases *CHIT5* and *CHIT5*-like (LOC115724705 and LOC115695573), germacrene-A synthase (LOC115695573), and ferredoxin-NADP reductase embryo like (LOC115724444) but did not exhibit clear relationships between histone marks H3K4me3 and H3K56ac and transcription. The chromatin state of germacrene-A synthase (LOC115695573) was indistinguishable between either tissue at the epigenomic level, except for more pronounced H2A.Z deposition near the promoter region. Ferredoxin-NADP reductase embryo-like (LOC115724444) was expressed solely in the glandular trichomes, yet there was no discernible difference in either H3K4me3 or H3K56ac deposition between glandular trichomes and other tissues. There was however enrichment of H2A.Z in the gene body of LOC115724444 in glandular trichomes. Furthermore, throughout the length of BGC #28 there was more pronounced enrichment of H2A.Z in trichomes comparatively, a feature which has been associated with tissue specific BGC regulation in *A. thaliana* (Additional File 1: Fig. S9b) [65].

We also observed a novel putative cluster of six aromatic prenyltransferases with trichome specific histone marks and gene expression (Fig. 4b, Additional File 7). This included the cannabigerolic acid synthases PT1, PT4, and PT7 (LOC115713215, LOC114713185, and LOC115713205) cannaflavin biosynthesis gene PT3 (LOC115713148) as well as two additional uncharacterised CBGAS-like genes (LOC115713171 and LOC 115722991) in a 347 Kbp region of the X chromosome [66, 67]. These genes had glandular trichome specific enrichment of H3K4me3 and H3K56ac in their promoters compared to other intervening genes, consistent with their active transcription in that tissue.

### Identification of putative glandular trichome specific regulatory motifs using H3K56ac enrichment

**Fig. 5:**
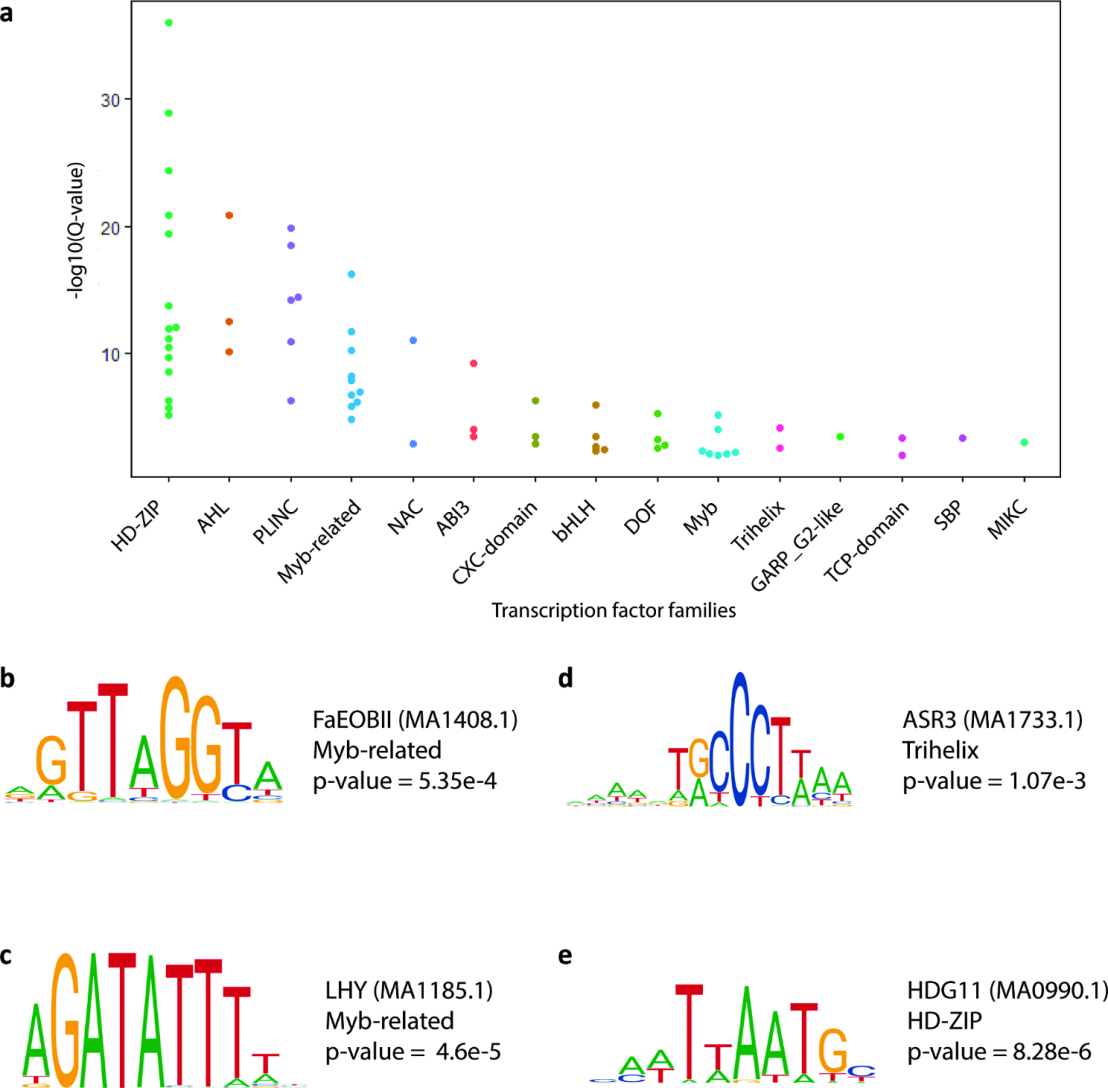
Putative glandular trichome specific cis regulatory elements are enriched in transcription factor binding motifs involved in specialised metabolism, circadian rhythm, and stress resistance. (**a**) Plot showing various transcription factor families associated with mined motifs in glandular trichome specific cis-elements. Each point represents the enrichment significance score of a transcription factor motif found in our dataset, plotted above its cognate family. (**b**) FaEOBII (MA1408.1) motif enriched in putative trichome specific cis-elements is associated with aroma volatile production. (**c**) LHY (MA1185.1) and CCA1 (MA0972.1) motifs involved in circadian rhythm homeostasis is enriched in trichome specific cis elements. (**d**) The pattern triggered immunity related ASR3 (MA1733.1) motif and (**e**) negative regulator of trichome branching associate regulatory motif HDG11(MA0990.1) are enriched in trichome specific cis regulatory elements

Cis-regulatory elements (CREs) are features within genomes which contain DNA sequence motifs that TFs can bind to. TFs drive gene expression through interactions with target gene promoters, either because the CRE is within a promoter itself, or through long-range interactions between a promoter and an enhancer that contains the CRE. Active plant CREs are often flanked by H3K56 acetylated chromatin [29]. We reasoned that we could mine putative CREs with glandular trichome specific activity by identifying enriched DNA sequence motifs within H3K56ac loci specific to glandular trichomes. The 1,048 H3K56ac loci specific to glandular trichomes were significantly enriched for 65 DNA sequence motifs (q < 0.05, JASPAR CORE database). The enriched motifs included MYB, MYB-related, NAC, and TCP transcription factor binding motifs (Fig. 5a, Additional File 10).

We examined the cognate transcription factors that may recognise these motifs and their likely functions, to better understand how they may be related to glandular trichome biology. For example, the MYB-related *Fragaria ananassa* emission of benzoid II (FaEOBII) cis element was enriched in putative CREs (Additional File 10). The corresponding best blast P hit for its associated transcription factor in the cs10 reference assembly was the MYB-related transcription factor CsEOBI (LOC115699015). CsEOBI is highly expressed in the glandular trichomes compared to either leaf (log2FC = 7.49, BH p.adj = 0.000105) or stem (log2FC = 8.08, BH p.adj = 1.21E-05). FaEOBII is known to positively regulate production of volatile organic compounds (Fig. 5b) [68]. Interestingly, LHY and CCA1 motifs were also enriched in putative CREs. The cognate transcription factors for these motifs work cooperatively to regulate circadian rhythms (Fig. 5c) [69]. The trihelix family member ASR3 motif was enriched, and its associated transcription factor negatively regulates pattern triggered immunity (PTI) that mitigates unnecessary hypersensitivity responses (Fig. 5d). HDG11 motifs were also enriched in glandular trichome specific H3K56ac chromatin. HDG11 has been shown to negatively regulate trichome branching in other plant species (Additional File 10).

### Predicting individual enhancer-gene interactions specific to glandular trichomes

Enhancer elements regulate gene expression by long range interactions with gene promoters, over kilobases to megabases. Enhancers contain CREs within which TFs bind, driving the long-range interactions, and so often carry H3K56ac histone marks. We therefore identified putative enhancer-gene target pairs active in glandular trichomes by making use of our CRE analysis results. We focused only on H3K56ac loci greater than 1.5 Kbp from the nearest TSS, to exclude gene-proximal promoters. These H3K56ac loci were then matched with the nearest gene that was expressed specifically in glandular trichomes, identifying 87 putative enhancer-target gene pairs (Table 1, Additional File 11). Strikingly, this strategy captured H3K56ac loci associated with the expression of hallmark glandular trichome associated genes. These included genes involved in terpenoid specialised metabolism, such as sesquiterpene biosynthesis; alpha-humulene synthase (LOC115724563), monoterpene biosynthesis; myrcene synthase (LOC115716405) and limonene synthase (LOC115716064), and triterpene biosynthesis; beta-amyrin synthase (LOC115719748). They also included genes involved in cannabinoid specialized metabolism, such as biosynthesis of the cannabinoid precursor hexanoate Butanoate-CoA ligase AAE1 (LOC115713865), as well as cannabidiolic acid synthase (LOC115697762). Fatty acid biosynthetic genes implicated in the upstream production of fatty alkyl side groups, required for cannabinoid production, were also found (Lineolate 13 S-lipoxygenase, LOX1, LOC115719612; Delta (12)-oleate desaturase, FAD1, LOC115719329), as were specialised metabolite transporters (ABC transporter B family member 2, LOC115716265; ABC transporter G family member 20, LOC115711415), and a transcription factor involved in trichome maturation (MYB106, LOC115701410). Interestingly, several of the enhancer-gene target predictions identified genes involved in biotic stress resistance that have not yet been reported in studies of glandular trichomes, including disease resistance protein RMP1 (LOC115704534) that is currently being investigated in wheat for its properties in resistance to powdery mildew, and an unusual protease neprosin (LOC115720846) found in carnivorous plants.

**Table 1:** Closest neighbouring genes that are expressed glandular trichome specifically to *C. sativa* glandular trichome specific gene distal H3K56ac loci, showing expression – a list of putative tissue specific enhancer gene interactions. * indicates intragenic putative cis-regulatory elements

Category	Gene ID	Description	Relative expression trichome/stem		Relative expression trichome/leaf		Putative enhancer -H3K56ac locus	Refs.
			log2FC	*p*.adj	log2FC	*p*.adj		
Cannabinoid biosynthesis	LOC115697762	Cannabidiolic acid synthase (CBDAS)	7.32	2.24E-09	9.15	7.3E-09	Chr7 30,985,961 − 30,986,744	[70, 71]
	LOC115697886	Cannabichromenic acid synthase-like	8.174	7.38E-06	7.58	6.8E-05	Chr7 26,086,586 − 26,087,174	
Sesquiterpenoid biosynthesis	LOC115724563	Alpha-humulene synthase	4.841	1.63E-19	7.145	3.90E-31	Chr6 78,980,722 − 78,981,538	[72]
Triterpene biosynthesis	LOC115719748	Beta-amyrin synthase	9.13	5.42E-09	8.54	1.23E-07	Chr2 81,831,322 − 81,832,508	[73]
Monoterpenoid biosynthesis	LOC115716064	(-)-Limonene synthase	7.595	4.12E-28	11.28	1.98-E48	Chr5 2,427,955 -2,429,181*	[74]
	LOC115716405	Myrcene synthase	6.792	2.17E-13	8.807	2.17E-13	Chr5 2,520,951 -2,522,151*	
Aroma volatile biosynthesis	LOC115717307	Methionine gamma-lyase	4.84	9.23E-43	5.464	1.7E-56	Chr5 86,422,670 − 86,424,161	[75]
	LOC115718871	Pyruvate decarboxylase 1-like	7.886	3.53E-57	12.88	9.72E-20	Chr2 5,491,001–5,491,980	[76]
	LOC115716705	branched-chain amino acid aminotransferase 1	5.107	7.07E-13	7.384	1.62E-07	Chr5 7,467,915 -7,469,631*	[77]
Hexanoate biosynthesis	LOC115713865	Butanoate—CoA ligase (AAE1)	2.191	0.000929	2.955	4.3E-09	ChrX 70,642,217 − 70,643,833	[78–81]
	LOC115695656	2-alkenal reductase (NADP (+) dependent)	6.654	1.34E-23	10.656	1.55E-12	Chr6 17,801,253 − 17,802,062*	
Oxylipin pathway	LOC115719329	Delta (12)-oleate desaturase (FAD1)	6.335	1.63E-39	9.606	3.56E-64	Chr2 87,098,771 − 87,099,548	
	LOC115719612	Lineolate 13 S-lipoxygenase (LOX1)	5.03	1.45E-33	2.148	0.0048	Chr2 93,912,176 − 93,919,286	
Fatty acid biosynthesis	LOC115705717	3-ketoacyl-CoA synthase 19	3.946	6.33E-08	8.833	1.87E-10	Chr1 22,744,264 − 22,745,167*	[82, 83]
Desaturase	LOC115724633	Fatty acid desaturase 4-like 1	6.688	1.86E-46	9.642	1.62E-25	Chr6 70,113,632 − 70,114,540	[84]
Protease	LOC115720846	Protein neprosin	4.43	1.26E-18	4.52	1.75E-17	Chr2 12,637,583 − 12,638,999	[85]
Powdery mildew resistance	LOC115704534	Disease resistance protein (RMP1)	9.23	3.47E-10	9.61	6.85E-11	Chr1 89,873,411 − 89,875,839*	[86, 87]
Innate immunity	LOC115722088	Epsin-3	8.157	2E-06	4.48	9.7E-05	Chr9 142,307 − 143,004	[88]
Metabolite transport	LOC115716265	ABC transporter B family member 2	4.133	1.13E-20	3.258	9.61E-11	Chr5 83,485,739 − 83,487,630*	[89]
	LOC115711415	ABC transporter G family member 20	5.18	8.92E-17	4.39	1.92E-11	Chr3 66,573,005–66,573,376	[90, 91]
Trichome maturation	LOC115701410	MYB106	4.93	2.97E-59	3.64	6.63E-34	Chr8 62,597,900 − 62,598,481	[92]
Phloem protein	LOC115723346	Sieve element occlusion B	6.46	4.93E-43	8.21	2.3E-52	Chr9 32,017,566 − 32,018,467*	[93]

## Discussion

*C. sativa* is a plant with significant biotechnological potential owing to its specialised metabolite productivity from large capitate stalked glandular trichomes. Despite our reliance on plant specialised metabolite products in industry, food, and medicine, our understanding of glandular trichomes is sparse. Currently, only a single publication in plants investigates epigenomic regulation of plant glandular trichome activity [94]. Here, we have provided the first H3K4me3, H3K56ac, H3K27me3, and H2A.Z chromatin maps in *C. sativa* glandular trichomes, and in plant glandular trichomes more broadly. Additionally, we demonstrate that classical eukaryotic functions of histone post translational modifications are functionally conserved in the *C. sativa* epigenome (Fig. 1).

Our chromatin landscape maps have been assembled using gold standard ENCODE guidelines to provide confidence in the observations made in this study. We provide these as a web browser resource for easy data re-use and for readers to inspect individual loci of interest (https://jbrowse.latrobe.edu.au/cannabis_trichome_epigenome/). A key theme we observe in this study is that chromatin modifications often co-localise, presumably to co-operatively regulate gene expression. For example, H3K56ac and H3K4me3 often co-localize at the promoters and TSSs of actively transcribed genes, whilst H3K27me3 and H2A.Z co-localize in the gene bodies of silenced genes (Fig. 2).

Our analysis highlights tissue-specific gene expression and chromatin states consistent with focal points of specialised metabolism, carbon trafficking, and plant abiotic and biotic stress resistance, as would be expected given the functions of glandular trichomes. For example, we observed that glandular trichome specific putative H3K4me3-H3K27me3 bi-valent chromatin is enriched in specialised metabolism, defence signalling, and innate immunity associated genes. We speculate that complex chromatin landscapes like these have the potential to integrate environmental cues to dynamically allocate carbon resources as needed during defence response elicitation like that reported in camalexin biosynthesis (Fig. 3) [53]. It should be noted, however, that overlaps between H3K4me3-H3K27me3 loci in leaf or stem tissue might reflect the heterogenous composition of those tissues, as opposed to a bona-fide signal of bi-valency. This limitation is less probable in the glandular trichomes samples, which were isolated to a high degree of purity largely composed of disc cells.

Previous work in *A. thaliana* suggests unique chromatin landscapes regulate plant BGC expression, so examining these landscapes provides an opportunity to better define *C. sativa* BGCs and understand how their activity may be regulated [95, 96]. We investigated our previously predicted putative *C. sativa* BGCs for evidence of trichome specific gene expression and chromatin composition [64]. We observed that two putative BGCs (#17, terpene-related; #28, terpene and fungal resistance related) were expressed specifically in glandular trichomes. Most genes in both BGCs displayed enrichment of promoter H3K4me3 and H3K56ac specifically in glandular trichomes, consistent with active gene expression. Observations made in *A. thaliana* suggest enrichment of H2A.Z is functionally associated with active BGCs, and enrichment of H3K27me3 at those BGCs in tissues where they are not expressed [97]. Contrasting with this, we did not observe enrichment of H2A.Z in a tissue specific manner that might explain trichome specific expression of either BGC. However, we did observe enrichment of H3K27me3 in BGC #17 monoterpene genes in stem and leaf tissue, where these genes were not expressed. Nonetheless, our data prompts further research into the association of distinct H2A.Z and H3K27me3 chromatin states at active and repressed BGCs. Future investigation might also consider the capacity of H2A.Z, a histone variant, to be post-translationally modified itself [44].

We made use of the association between H3K56ac and CREs to identify putative *C. sativa* glandular trichome specific TFs and binding motifs [29, 30, 98]. This strategy yielded candidates involved in aroma volatile production associated (FaEOBII, JASPAR ID MA1408.1), trichome development (HDG11; MA0990.1), and circadian rhythms (LHY; MA1185.1, CCA1; MA0972.1). Daytime progression has recently been implicated in cannabinoid production which might explain the enrichment of circadian rhythm associated motifs in our dataset (Fig. 5) [99]. Furthermore, we hypothesised that we could leverage our H3K56ac and gene expression datasets to mine the glandular trichome epigenome for putative enhancer-gene target pairs. This yielded several putative enhancer-gene pairs where the genes have known glandular trichome specific functions, including CBDAS (LOC115697762), terpenoid synthases (LOC115724563, LOC115719748, LOC115716064, LOC115716405), and metabolite transporters (LOC115716265, LOC115711415) [70, 91, 100, 101]. We note that our approach would favour identification of interactions at kilobase scale and would not allow us to identify enhancer elements operating at very long range, over intervening genes. Further study of these would require assays like Hi-C to capture the full scope of enhancer-target gene interactions. Nonetheless, the putative enhancer-gene interactions we identify here in *C. sativa* presents a significant biotechnological resource that may provide a framework for engineering glandular trichome gene expression in the future.

## Conclusion

In this study we provide novel insights into the chromatin landscape of glandular trichomes. Our work shows how chromatin features and function are conserved in *C. sativa* compared with more deeply characterised plant species, from feature types to correlation with gene expression, deposition and localisation within genes, and association with distal regulatory regions. Moreover, we show the advantages of studying such chromatin features where, for example, genes postulated to exist in previous studies have been predicted by our multimodal approach, and genome wide sequence information can be mined in tissue specific manners to identify cis-regulatory elements not yet observed in *C. sativa*. This work has laid a solid foundation for our understanding of glandular trichome chromatin dynamics with respect to tissue specific gene expression and cis-regulatory elements, and may to glandular trichome metabolic engineering efforts in years to come.

## Methods

### Plant material and growth conditions

Cuttings were taken from female genotype (MW6-15) [81]. MW6-15 was derived from an industrial hemp line (accession #6) previously donated to the university by Southern Cross University [102]. Cuttings were made using a sterile blade and at a 45-degree angle immediately below the 3rd node from the apical meristem. The cuttings were partially pruned – removing leaf tips and large leaves, taking care not to remove all leaves. The cuttings were then dipped in 3.0 g/L indole butyric acid rooting gel hormone (Growth Technology © Clonex purple) and allowed to sit for 30 s. Cuttings were individually transferred to Grodan Rockwool cubes (36 mm x 36 mm x 40 mm) previously saturated with half strength CANNA veg fertiliser (20 mL A + 20 mL B/ 10 L water), and then transferred to propagator humidity domes (4 clones to one propagator) with the vent fully closed. Clones were monitored daily – half strength CANNA veg fertiliser (20 mL A + 20 mL B/ 10 L water) was added to the base of the propagator when required and vents were opened in small daily increments until completely opened and grown under vegetative lighting conditions of 18 h on 6 h off using Philips Master TL-D Super 80 low-pressure mercury discharge lamps. Clones were kept in propagators until established roots could be observed (approximately 2 weeks) at the base of the rockwool cubes. Potting mix containing 1:1:1 peat moss, perlite, and vermiculite supplemented with 1 g/L dolomite was prepared and divided into 1 L pots. Pots were then saturated with full strength CANNA veg fertiliser (40 mL A + 40 mL B/ 10 L water). Clones were then transferred to appropriately labelled 1 L pots and allowed to grow under vegetative lighting conditions for 4 weeks. Plants were monitored daily and watered regularly using full strength CANNA veg during the vegetative growth stage. Following 4 weeks of vegetative growth plants were re-potted into appropriately labelled 10 L pots using the above potting mix recipe. Potting mix was saturated with full strength CANNA flora (40 mL A + 40 mL B/ 10 L water) prior to re-potting of the clones. The lighting cycle was then varied to 12 h on and twelve hours off to induce flowering. Clones were grown under flowering conditions for 6 weeks and watered daily with full strength CANNA flora. Clones were harvested during the late stage of flowering as defined by a colour change, from white to brown, of greater than two thirds of all stigmas on the plant.

### Tissue harvest and trichome isolation for RNA sequencing

For RNA extraction, fresh samples were collected in three biological replicates from mature fan leaves, 3 cm internodal stem segments, and female inflorescences. Triplicates were obtained from three individual plants. Trichomes were isolated from female inflorescences using a method modified from a protocol previously described [103]. Briefly, ~ 5 g samples were transferred to 50 mL Falcon™ tubes and about 10 mL of liquid nitrogen was added to each tube. The tubers were loosely capped and vortexed until the trichomes were fully removed. After removing plant debris by inverting and tapping the tubes, trichome-enriched samples were carefully transferred to 2 mL Eppendorf tubers in liquid nitrogen for further processing.

### Tissue harvest and trichome isolation for chromatin immunoprecipitation

Samples of stem (internode) and vegetative leaves were taken from each clone using a sterile blade. Each sample was transferred to an appropriately labelled 50 mL falcon tube, sealed, and immediately flash frozen in a Dewar containing liquid nitrogen. Samples were then stored at -80 °C.

Glandular trichomes were isolated by ice-water extraction – all inflorescences were harvested from an individual clone and cut into small 5 cm x 5 cm pieces using sterile secateurs. Nylon bags with 25 μm, 45 μm, 73 μm, 120 μm, and 160 μm mesh sizes were sequentially placed inside one another starting the smallest, outermost, 25 μm mesh bag. The setup was then placed in a 5 L beaker and then filled with 1:3 ice-cold milli-q water: crushed ice. The cut inflorescences were then added to the beaker/nylon-mesh set-up and stirred using a large metal spatula for ten minutes. The nylon-mesh bags were then removed from the beaker and the contents of the beaker were then filtered through a nylon mesh with 25 μm pore size. Glandular trichomes were then retrieved, *via* pipetting, from the surface of the 25 μm nylon mesh and transferred into appropriately labelled falcon tubes. Glandular trichomes were immediately frozen in liquid nitrogen and then transferred to cold storage at -80 °C.

### Tissue preparation and chromatin cross-linking

Glandular trichomes, stem (internode), and vegetative leaves were retrieved from cold storage (-80 °C) and approximately 1 g of each tissue was ground into a fine powder using liquid nitrogen and a pre-chilled mortar and pestle. The ground tissues were then transferred into appropriately labelled 15 mL falcon tubes followed by the addition of 12.5 mL of nuclear isolation/ cross-linking buffer (60 mM HEPES at pH 8.0, 1 M Sucrose, 5 mM KCl, 5 mM EDTA at pH 8.0, 0.6% (v/v) Triton X-100, 1 mM PMSF (Sigma-Aldrich 93482-50ML-F), 1 mM pepstatin A (Sigma-Aldrich P5318-5MG), 1 mini-complete tablet (Sigma-Aldrich 11836170001) per 10 mL buffer) on ice (Additional File 12: Table A1). The contents of each tube were stirred until a homogenous suspension had formed. Next, 360 µL of 37% formaldehyde was added to each of the sample suspensions - cross-linking was achieved through incubation for 25 min at room temperature and gentle rotation. Cross-linking was halted through the addition of 875 µL 2 M glycine to each of the sample suspensions followed by 25 min incubation at room temperature and gentle rotation.

### Nuclei isolation

Nuclei were isolated from the sample suspensions through passive filtration into a 50 mL falcon tube using a 40 μm nylon mesh sieve. The filtrate was then centrifuged at 4000 RPM for 20 min at 4 °C. The supernatants were carefully removed, and the soft nuclei pellets were resuspended, by pipetting, using 1 mL of extraction buffer (0.25 M Sucrose, 10 mM Tris-HCl at pH 8.0, 10 mM MgCl2, 1%(v/v) Triton X-100, 1 mM EDTA at pH 8.0, 5 mM β-mercaptoethanol, 1 mM PMSF, 1 mM Pepstatin A, 1 mini-complete table per 10mL) (Additional File 12: Table A2) and then transferred to a 2 mL Eppendorf tube. The walls of each 50 mL falcon tube were washed twice with 100 µL of extraction buffer and transferred to its corresponding Eppendorf tube to collect any residual nuclei. Tubes were then sealed and centrifuged at 11.4k RPM for 10 min at 4 °C and the supernatant discarded.

### Chromatin shearing

Each of the pellets were then resuspended in 300 µL of nuclei lysis buffer (50 mM Tris-HCl at pH 8.0, 10 mM EDTA at pH 8.0, 1% (w/v) SDS, 1 mM PMSF, 1 mM Pepstatin A, 1 mini-complete table per 10 mL buffer) (Additional File 12: Table A3). Samples were then sonicated using a Diagenode Bioruptor^®^ on high setting, 30 s on, 30 s off, for 15 min at 4 °C. Samples were then centrifuged at 5000 RPM for 10 min at 4 °C to pellet unwanted cellular/nuclear debris. The supernatant, containing sheared chromatin, was then transferred to new Eppendorf tubes on ice. All samples were then placed in cold storage at -80 °C.

### Chromatin immunoprecipitation

In preparation for immunoprecipitation 60 µL of Dynabeads-Protein A (Thermo Fisher Scientific, #10002D) per sample per histone mark was washed with 30 µL of ChIP dilution buffer (1.1% (v/v) Triton X-100, 1.2 mM EDTA at pH 8.0, 16.7 mM Tris-HCl at pH 8.0, 167 mM NaCl, 1 mM PMSF, 1 mM Pepstatin A, 1 mini-complete tablet per 10 mL buffer) (Additional File 12: Table A4). Samples were pre-cleared by adding 90 µL of the washed Dynabeads-Protein A and incubating for 4.5 h, rotating, at 4 °C. Eppendorf tubes were labelled with the appropriate sample name and histone mark and 60 µL aliquots of the washed Dynabeads-Protein A were made into each tube followed by the addition of 2.5 µL (2.5 µg) of either Anti-H3K4me3 (Millipore^®^, 07-473), Anti-H3K56ac (Millipore^®^, 07-677-1), Anti-H3K27me3 (Millipore^®^, 07-449), or Anti-H2A.Z (Millipore^®^, 07-594) to their correspondingly labelled tubes. The tubes were then sealed and incubated at 4 °C, rotated, for 1 h.

Following pre-clearing, samples were then placed on a magnetic stand (Thermo Fisher Scientific, #AM10027) for 2 min until all Dynabead-Protein A precipitate out of solution. Next, 300 µL of the supernatant, of each tissue type, was then transferred to a new tube to use as an input control. One input control was made for each of the tissue types used in the experiment. The INPUT controls were then immediately placed in storage at -20 °C. The remaining 3.6 mL of supernatant was then made into 4 × 900 µL aliquots and transferred to the previously labelled, tubes, containing the Dynabeads-Protein A/antibody slurry and incubated at 4 °C, rotating, for 90 min. The samples were then placed on a magnetic stand for 2 min and the supernatant was removed. On the stand, 1 mL of low salt wash buffer (150 mM NaCl, 0.1% (w/v) SDS, 1% (w/v) TritonX-100, 2 mM EDTA at pH 8.0, 20 mM Tris-HCl at pH 8.0) was added to each tube (Additional File 12: Table A5). The tubes were then sealed, and the contents were resuspended by inverting and then placed on the magnetic stand for 2 min until all the Dynabeads-Protein A/antibody/chromatin complexes precipitate out of solution and the supernatant discarded. The wash step was repeated one more time using the low salt wash buffer, then again using the high salt wash buffer (500 mM NaCl, 0.1% SDS, 1% TritonX-100, 2 mM EDTA at pH 8.0, 20 mM Tris-HCl at pH 8.0) (Additional File 12: Table A6), followed by a final wash using the LiCl wash buffer (0.25 M LiCl, 1% (v/v) NP-40 (Sigma-Aldrich NP40S-100 mL), 1% Sodium Deoxycholate, 1 mM EDTA at pH 8.0, 10 mM Tris-HCl at pH 8.0) (Additional File 12: Table A7). The beads were then washed using 1 mL of TE buffer (10 mM Tris-HCl at pH 8.0, 1 mM EDTA at pH 8.0) (Additional File 12: Table A8), allowed to precipitate on the magnetic stand for 2 min, followed by aspiration of the supernatant. Each sample was then removed from the magnetic stand and the pellets were resuspended with 150 µL of SDS elution buffer (1% SDS, 0.1 M NaHCO_3_) (Additional File 12: Table A9) and incubated at 65 °C for 15 min. The samples were then placed on the magnetic stand for 2 min and the supernatant was then transferred to a newly labelled 1.5 mL tube.

A second elution of the beads was performed using 150 µL of SDS elution buffer. The supernatants were combined in 1.5 mL tube for a final volume of 300 µL. A master mix solution containing 12 µL 5 M NaCl, 30 µL Dithiothreitol (DTT), and 30 µL 1 M NaHCO3 per sample was made up for all samples, including INPUT controls. 72 µL of the master mix was aliquoted into each of the samples and INPUT controls and allowed to incubate overnight at 65 °C. Samples and INPUT controls were retrieved from overnight incubation and 6 µL 0.5 M EDTA, 12 µL 1 M Tris pH 7.0, and 2 µL proteinase K was added to each tube. Tubes were then incubated at 45 °C for 1 h. Samples and INPUT controls were then cleaned up by adding 350 µL chloroform/ isoamyl alcohol (24:1), vortexing, and centrifuging using a bench top centrifuge at max speed for 25 min at room temperature. The top layer was then carefully, without disturbing the interphase, transferred to a new tube. 2 µL of glycogen, 60 µL of 3 M sodium acetate pH 5.2, and 900 µL of chilled ethanol (95%) was added to each sample and INPUT control and then allowed to precipitate overnight at -20 °C. Samples and INPUT controls were then centrifuged at 16,000 g, for 30 min, at 4 °C. The DNA pellet was then washed two times using 1 mL of ethanol (70%). The washed DNA pellets were then allowed to dry in a fume hood. The dry DNA pellets were then resuspended in 50 µL TE buffer. Double stranded DNA (dsDNA) content was quantified with the Qubit dsDNA high sensitivity assay using the Qubit 4 fluorometer (See Additional File 1). ChIP-seq libraries were then generated using the Accel-NGS 2 S Plus DNA library Kit (Swift biosciences), following the manufacture’s recommendations using 10 pg – 250 ng of input dsDNA per sample.

### Assessing library quality – tapestation

The quality of the sequencing libraries was assayed using a 2200 Tapestation (Agilent) and D1000 screen tape. Samples were prepared using 1 µL of each sample library and diluted using 3 µL of D1000 sample buffer for a final volume of 4 µL. Tubes were then sealed and vortexed for 30 s. The samples were then spun down in a microfuge for 1 min to ensure residual droplets on the tube walls were collected at the base of each tube. Sample were then run on the Tapestation, and the results recorded.

### Chromatin immunoprecipitation sequencing operations

Samples were sequenced on an Illumina next-seq according to manufacturer’s instructions. We applied the ENCODE consortium guidelines for broad (H3K37me3, H3K56ac, H2A.Z) and narrow peak (H3K4me3) type data in humans of > 45 million reads and > 20 million reads are recommended respectively. For *C. sativa* the target reads were linearly scaled approximately 4 times to > 10 million reads and > 5 million reads respectively to account for the genome size discrepancy between human and *C. sativa*.

### RNA sequencing library preparation and sequencing operations

All samples were homogenised using Geno/Grinder 2010 (SPEX SamplePrep) and total RNA was isolated from homogenised samples using the Sigma Spectrum Plant Total RNA kit (Sigma) supplemented with the On-Column DNase I Digestion step to remove genomic DNA. RNA was eluted in 50 µL EB buffer. RNA concentration was measured using a Nanodrop spectrophotometer. RNA-seq library generation was performed using Illumina TruSeq Stranded mRNA Library Prep and indexed using Truseq RNA UD Indexes (96 indexes, 96 samples). Individual libraries were quality checked using Qubit dsRNA HS Assay kit and Agilent Tapestation (D1000) before being pooled into one sample for sequencing using Illumina NextSeq 500/550 High Output kit v2 (75 cycles).

### ChIP-seq data processing

Raw sequencing reads were quality trimmed using Trim Galore version 0.6.3 and the following parameters were used for paired end reads -paired -trim1 -fastqc [104]. The -fastqc option was selected to provide a quality report of the trimmed reads after trimming.

Quality trimmed reads were then aligned to the cs10 version 2 reference genome GCF_900626175.2 using Bowtie2 version 2.3.5.1 using the default parameters for paired end reads. Bowtie2 output SAM files were processed using Samtools version 1.9. SAM files were first converted to BAM files using the Samtools view -Sb function. Samtools view output BAM files were then sorted using the Samtools sort function [105]. The aligned reads were then filtered for PCR duplicates. Using the Picard version 2.2.2 suite of tools PCR duplicates were marked and subsequently removed from the aligned files using the MarkDuplicates function with the following parameters: TAG_DUPLICATE_SET_MEMBERS = true TAGGING_POLICY = All REMOVE_DUPLICATES = true ASSUME_SORT_ORDER = coordinate READ_NAME_REGEX = null INDEX = true.

### RNA-seq data processing

The quality of the raw RNA-seq data was assessed using FastQC v0.11.9 [106]. The data was aligned in single-end mode against the *Cannabis sativa* reference genome (cv. cs10; accession number GCF_900626175.2) using HISAT2 v2.1.0 (default parameters) [107]. The mapped reads were sorted using Samtools v1.9 and transcript per million (TPM) counts were generated using StringTie v2.1.3b [105, 108]. Gene-level quantification was performed on the mapped reads using featureCounts from the Subread v2.0.0 package [109]. Principal component analysis (PCA) plots were generated in R v4.1 using the normalised read counts from the DESeq2 v1.44.0 R package [110].

### Peak calling

Narrow peak type data was called for each biological replicate for the narrow peak type H3K4me3 and for the mixed peak type data H3K56ac using model based analysis of ChIP-seq (MACS2 2.2.7) using the parameters bam paired end function -f BAMPE and mappable genome size -g 736,579,359 -B and a relaxed p value cut off -p 0.1 as indicated for downstream irreproducible discovery rate (IDR) analysis [111]. Statistically significant, replicable peaks were then estimated using the irreproducible discovery rate (IDR 2.0.4.2) [112]. An IDR threshold or false discovery rate of 0.05 was applied for transcription factor-like H3K4me3 narrow peaks.

There was no community standardised approach for handling mixed peak types like H3K56ac. We consequently handled the dataset as a narrow peak type dataset, as we were primarily interested in the narrow domain functionality of H3K56ac [113]. As IDR is considered particularly stringent in calling reproducible peaks for narrow peak type data, and there is inherent broad domain noise in H3K56ac data, we increased the IDR or false discovery rate cutoff threshold to 0.1 for H3K56ac datasets.

The ENCODE consortium does not have guidelines on how to process biological replicates for H3K27me3 and H2A.Z broad domains. We consequently took a conservative approach to identifying reproducible broad domains between biological replicates for both H2A.Z and H3K27me3. We used spatial clustering for identification of ChIP-seq regions (SICER), epic2 0.0.52 specifically designed for broad domain peak type data using default parameters. Prior to epic2 calling of broad domains we scaled each replicate and its corresponding input control, as epic2 does not have auto-scaling functionality like MACS2 software, using the deepTools 3.5.1 bamcoverage option and --scalefactor parameter. We then identified the replicable broad domains between the replicates using BEDTools 2.3.0 intersect option requiring that broad domains share at least 30% reciprocal overlaps -f 0.3 -r.

### Gene and KEGG ontology analysis

To identify genes associated with various chromatin states we used BEDTools 2.3.0 intersect function to find genes genome-wide that overlapped with replicable peaks for each histone mark/modification. We then used these gene lists to perform Gene ontology and Kyoto encyclopedia of genes and genomes (KEGG) ontology analysis. We used the R-Package ClusterProfiler 4.0 [114] to perform gene ontology analysis using an in-house gene ontology database for the cs10 reference assembly (GCF_900626175.2) created using PANNZER2 [115]. Similarly, we used ClusterProfiler 4.0 to perform KEGG pathway enrichment using the available *C. sativa* KEGG ontologies for the cs10 reference assembly (GCF_900626175.2). For glandular trichome specific analysis we used BEDTools intersect -v function to report peaks that only occurred in glandular trichomes compared to stems and with that output we applied the same operation with the leaf dataset to identify glandular trichome specific peaks for each chromatin type. The genes intersecting the glandular trichome specific chromatin peaks were then subject to ontology analysis as above.

### Differential gene expression and gene set enrichment analysis

Differential gene expression was carried out on the RNA-seq datasets using the R-package DESeq2 with default parameters and selecting differentially expressed genes a > 2 log fold change and q-value > 0.05 [110]. The DESeq2 output results were then brought forward for gene set enrichment analysis using the ClusterProfiler 4.0 package ridgeplot function using the PANNZER2 cs10 (GCF_900626175.2) gene ontology database.

### Cis-regulatory motif analysis

Glandular trichome specific distal (≥ 1.5 Kbp from TSS) H3K56ac peaks were determined using the BEDTools 2.3.0 intersect -v function. The nucleotide sequences within peaks were extracted using the BEDTools 2.3.0 getfasta option. Similarly, the BEDTools 2.3.0 getfasta option was used to determine the nucleotide sequences of all glandular trichome H3K56ac peaks which were then used as background control for motif discovery. Motif discovery was carried out using MEME suite tools [116]. Glandular trichome specific H3K56ac chromatin sequences were enriched for motifs using the simple enrichment analysis (SEA) with default settings, cross-referencing the JASPAR CORE (2022) plants non-redundant motif database [116]. The significantly (q < 0.05) motifs were then plotted by transcription factor family vs. -log10(Q-value) using the beeswarm R-package.

### Distal enhancer and target prediction leveraging multiomic datasets

Using the list of glandular trichome specific H3K56ac peaks we then determined gene distal peaks occurring ≥ 1.5 kb from gene TSSs using the hypergeometric optimization of motif enrichment (HOMER) Perl script getDistalPeaks.pl using the parameters -gid -d 1500, then re-run using the parameter -targets to produce a list of the nearest gene-distal H3K56ac peaks [117]. We then used a custom python script to match the list of putative target genes to their corresponding DESeq2 differentially expressed gene ID in glandular trichome vs. stem and glandular trichome vs. leaf DESeq2 outputs. The list of genes was then filtered to include only those genes that show transcriptomic evidence of strong glandular trichome specific gene expression (log2FC > 2; p.adj < 0.05) in both trichome vs. stem and trichome vs. leaf datasets.

## Electronic supplementary material

Below is the link to the electronic supplementary material.

Supplementary Material 1

Supplementary Material 2

Supplementary Material 3

Supplementary Material 4

Supplementary Material 5

Supplementary Material 6

Supplementary Material 7

Supplementary Material 8

Supplementary Material 9

Supplementary Material 10

Supplementary Material 11

Supplementary Material 12

## Data Availability

Chromatin immunoprecipitation sequencing data was made publicly available on the sequencing read archive (SRA) under BioProject ID PRJNA1128358 complimentary RNA sequencing datasets were registered under BioProject ID PRJNA1128734. Web browser containing all alignments, peak calls, peak replicability, and differential epigenomic analysis https://jbrowse.latrobe.edu.au/cannabis_trichome_epigenome/.
