# Supplementary material for: Characterization of the Cannabis sativa glandular trichome epigenome
Source: BMC Plant Biol. 2024 Nov 14;24:1075. doi: 10.1186/s12870-024-05787-x (PMC11562870; doi:10.1186/s12870-024-05787-x)
Supplement: Supplementary file 1 — Supplementary Material 1 [file 12870_2024_5787_MOESM1_ESM.pdf]

Additional File 1. Supporting Figures and Tables

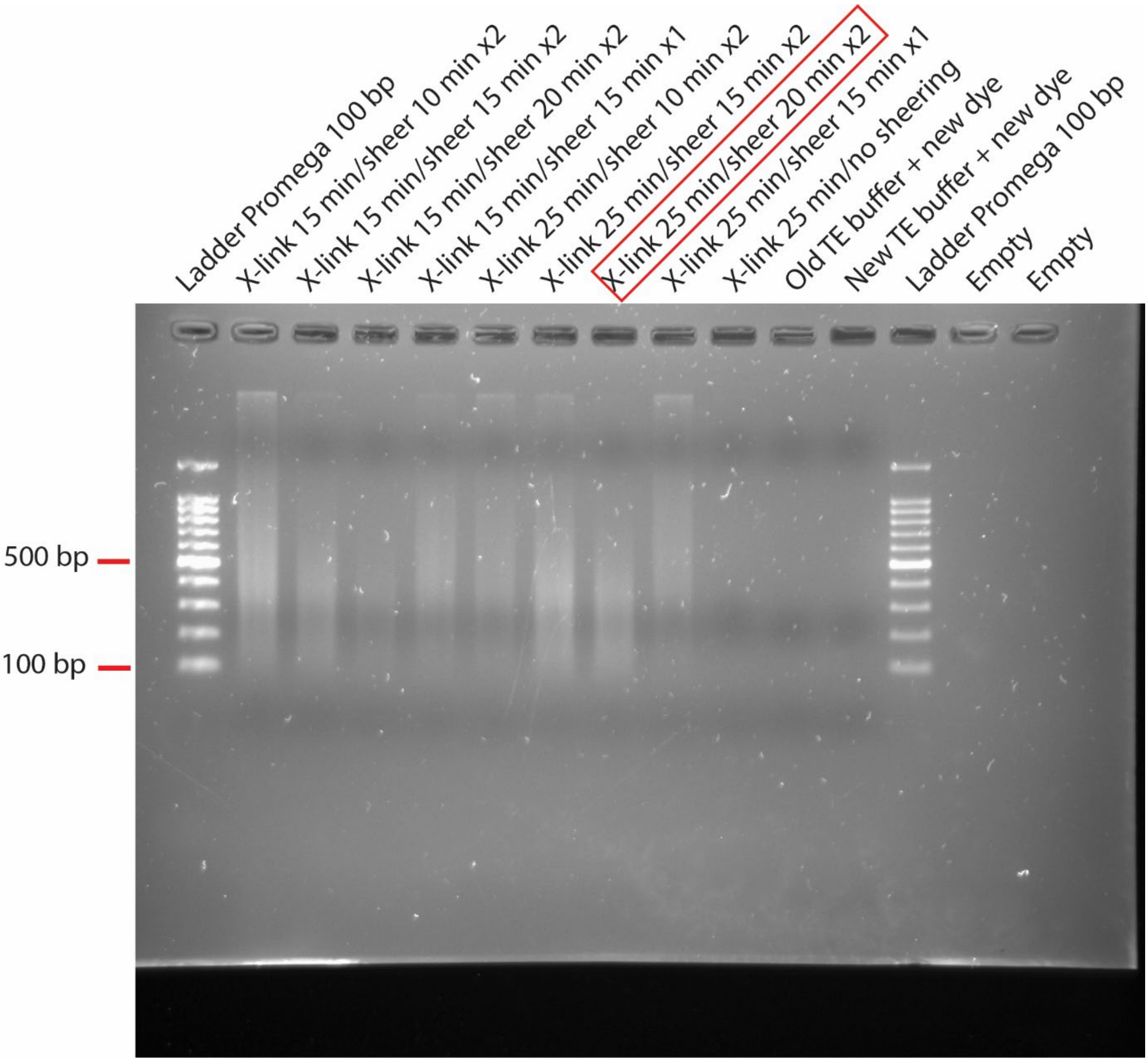

Figure S1. Optimization of Chromatin fragmentation conditions. Chromatin from *Cannabis sativa* was subjected to various sonication times and cycles under different cross-linking times to choose the most suitable strategy of obtaining chromatin fragments in the target 100 – 500 bp range. A final cross-linking time of 25 mins in combination with 2 cycles of 20 min sheering on high setting was selected for downstream immunoprecipitation.

Table S1. Qubit dsDNA high sensitivity Assay results of immunoprecipitated chromatin from glandular trichomes, stalks, and vegetative leaves. dsDNA content was estimated for each Immunoprecipitate to ensure a minimum sufficient mass of >10 pg was available for downstream library preparation using the Swift ACCEL-NGS® 2S Plus DNA library prep kit.

**Sample ID**	**dsDNA concentration**	
	**Biological Replicate 1**	**Biological Replicate 2**
INPUT control (Trichomes)	8.96 ng/µL	4.22 ng/µL
INPUT control (Stalks)	3.22 ng/µL	3.06 ng/µL
INPUT control (Vegetative leaves)	4.7 ng/µL	4.90 ng/µL
H2A.Z (Trichomes)	0.014 ng/µL	0.0112 ng/µL
H2A.Z (Stalks)	0.0062 ng/µL	0.0066 ng/µL
H2A.Z (Vegetative leaves)	0.0051 ng/µL	0.0104 ng/µL
H3K4me3 (Trichomes)	0.14 ng/µL	0.0504 ng/µL
H3K4me3 (Stalks)	0.0850 ng/µL	0.0804 ng/µL
H3K4me3 (Vegetative leaves)	0.0574 ng/µL	0.0240 ng/µL
H3K56ac (Trichomes)	0.112 ng/µL	0.0778 ng/µL
H3K56ac (Stalks)	0.0188 ng/µL	0.0162 ng/µL
H3K56ac (Vegetative leaves)	0.0148 ng/µL	0.0148 ng/µL
H3K27me3 (Trichomes)	0.0062 ng/µL	0.0069 ng/µL
H3K27me3 (Stalks)	0.0108 ng/µL	0.0106 ng/µL
H3K27me3 (Vegetative leaves)	0.0050 ng/µL	0.0071 ng/µL

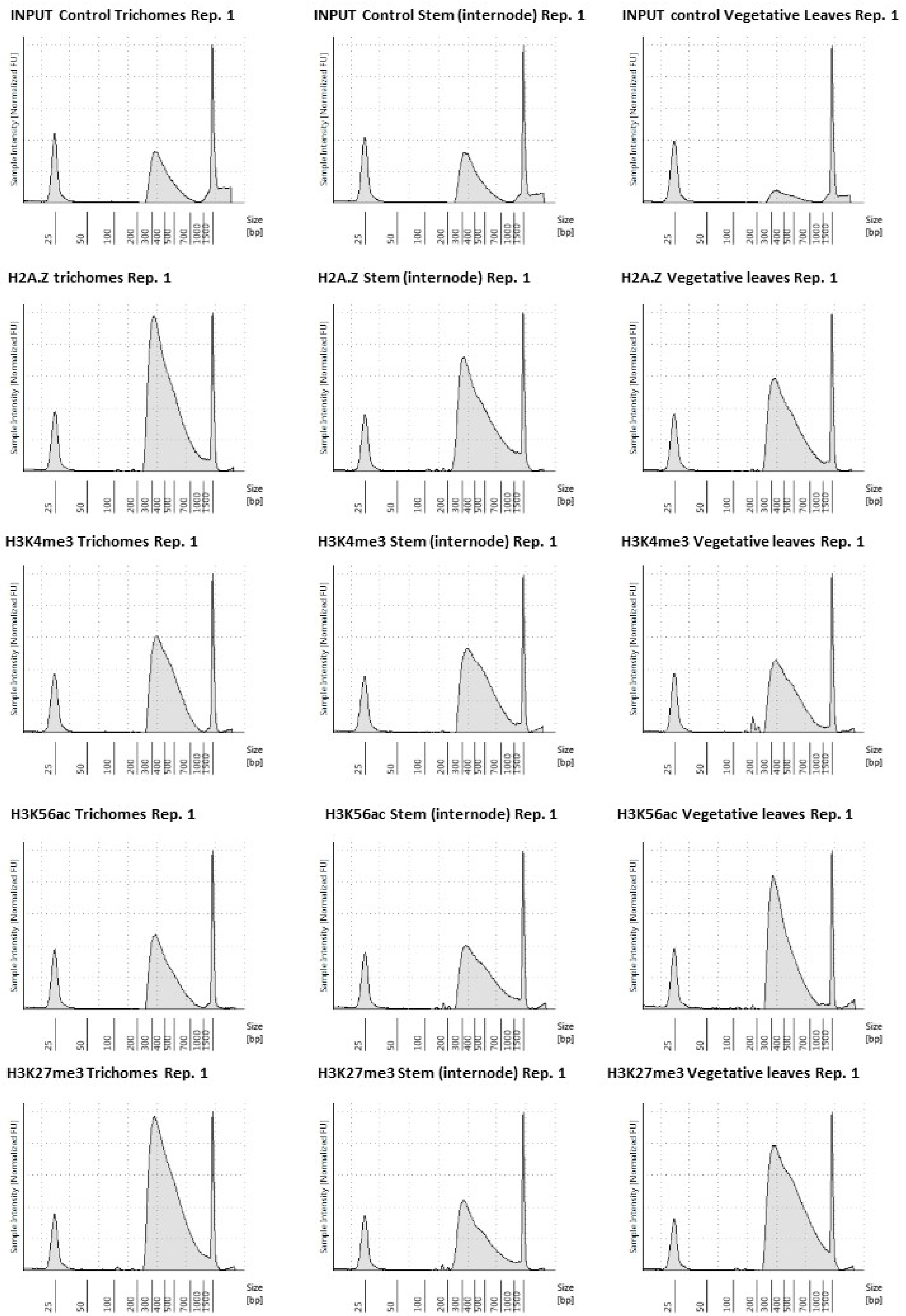
INPUT Control Trichomes Rep. 1
INPUT Control Stem (internode) Rep. 1
INPUT control Vegetative Leaves Rep. 1
H2A.Z trichomes Rep. 1
H2A.Z Stem (internode) Rep. 1
H2A.Z Vegetative leaves Rep. 1
H3K4me3 Trichomes Rep. 1
H3K4me3 Stem (internode) Rep. 1
H3K4me3 Vegetative leaves Rep. 1
H3K56ac Trichomes Rep. 1
H3K56ac Stem (internode) Rep. 1
H3K56ac Vegetative leaves Rep. 1
H3K27me3 Trichomes Rep. 1
H3K27me3 Stem (internode) Rep. 1
H3K27me3 Vegetative leaves Rep. 1
Sample Intensity [Normalized FU]
Size [bp]
25
50
100
200
300
400
500
700
1000
1500

INPUT Control Trichomes Rep. 2

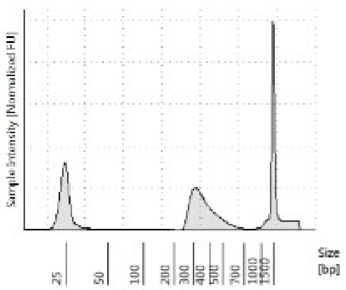

INPUT Control Stem (internode) Rep. 2

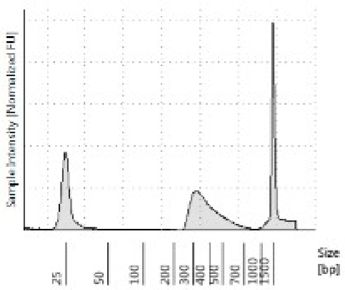

INPUT control Vegetative Leaves Rep. 2

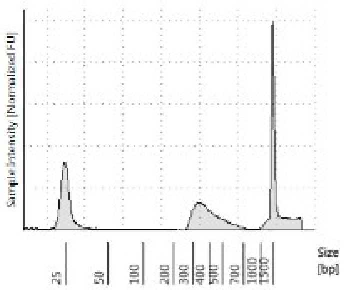

H2A.Z trichomes Rep. 2

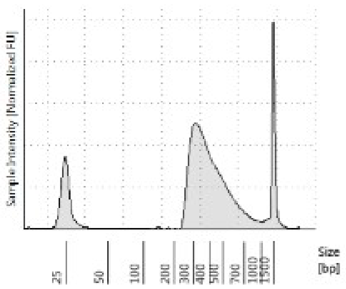

H2A.Z Stem (internode) Rep. 2

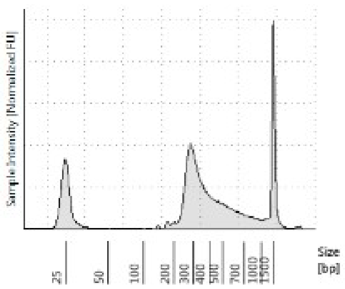

H2A.Z Vegetative leaves Rep. 2

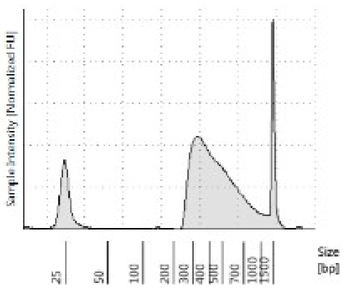

H3K4me3 Trichomes Rep. 2

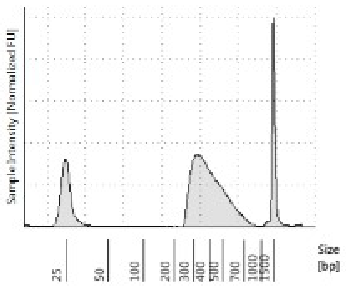

H3K4me3 Stem (internode) Rep. 2

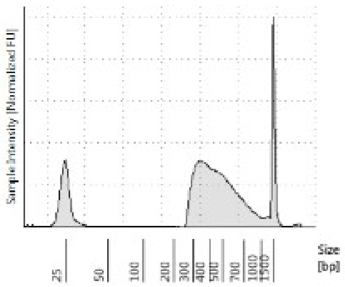

H3K4me3 Vegetative leaves Rep. 2

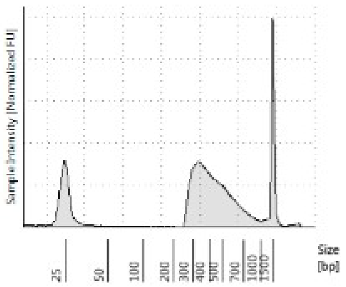

H3K56ac Trichomes Rep. 2

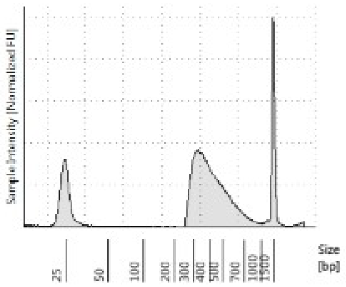

H3K56ac Stem (internode) Rep. 2

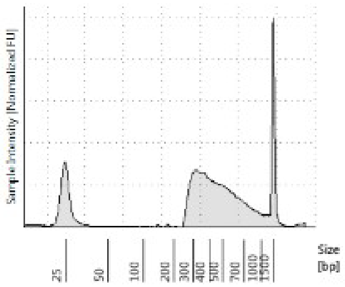

H3K56ac Vegetative leaves Rep. 2

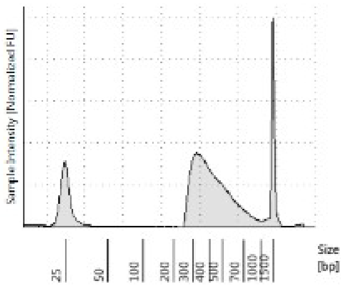

H3K27me3 Trichomes Rep. 2

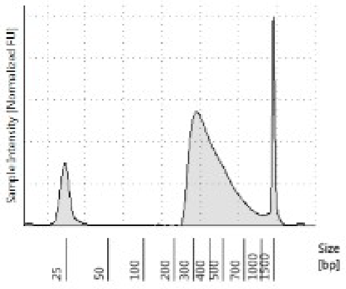

H3K27me3 Stem (internode) Rep. 2

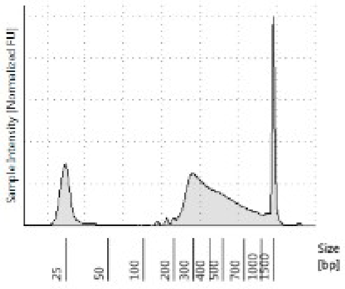

H3K27me3 Vegetative leaves Rep. 2

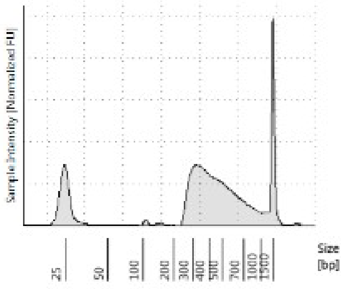

Figure S2. 2200 Tapestation D1000 electropherograms of ChIP-seq libraries. 2200 Tapestation D1000 electropherograms of 30 ChIP-seq libraries including INPUT control and 4 histone marks H2A.Z, H3K4me3, H3K56ac, and H3K27me3 for *Cannabis sativa* glandular trichomes, stem (internode), and vegetative leaves in biological duplicate.

Table S2. ChIP-seq libraries fragment size and concentration estimation. Average fragment size estimation from 2200 Tapestation D1000 results, and qubit concentration estimates for each library. Codes for Swift biosciences Accel-NGS 2S indexing adapters used for each library listed under the indexing adapters as well as the corresponding plate position.

Sample ID	Indexing adapter	Plate position	Average Fragment length bp	dsDNA ng/uL
INPUT control Trichomes Rep. 1	U037	A1	402	61.6
INPUT control Stem (internode) Rep. 1	U038	A2	393	59
INPUT control Vegetative leaf Rep. 1	U039	A3	422	62.2
H2A.Z Trichomes Rep. 1	U040	A4	430	67.4
H2A.Z Stem (internode) Rep. 1	U041	A5	452	52.2
H2A.Z Vegetative leaf Rep. 1	U042	A6	444	71.4
H3K4me3 Trichomes Rep. 1	U043	A7	431	80.2
H3K4me3 Stem (internode) Rep. 1	U044	A8	462	72.4
H3K4me3 Vegetative leaf Rep. 1	U045	A9	439	66.6
H3K56ac Trichomes Rep. 1	U046	A10	418	79.6
H3K56ac Stem (internode) Rep. 1	U047	A11	464	61
H3K56ac Vegetative leaf Rep. 1	U048	A12	410	71
H3K27me3 Trichomes Rep. 1	U049	B1	443	67.2
H3K27me3 Stem (internode) Rep. 1	U050	B2	434	58.2
H3K27me3 Vegetative leaf Rep. 1	U051	B3	470	71.5
INPUT control Trichomes Rep. 2	U061	G1	377	55.4
INPUT control Stem (internode) Rep. 2	U062	G2	319	57.2
INPUT control Vegetative leaf Rep. 2	U063	G3	410	48.8
H2A.Z Trichomes Rep. 2	U064	G4	420	53.4
H2A.Z Stem (internode) Rep. 2	U065	G5	410	45.2
H2A.Z Vegetative leaf Rep. 2	U066	G6	463	68.2
H3K4me3 Trichomes Rep. 2	U067	G7	415	60.2
H3K4me3 Stem (internode) Rep. 2	U068	G8	476	70.4
H3K4me3 Vegetative leaf Rep. 2	U069	G9	450	66.0
H3K56ac Trichomes Rep. 2	U070	G10	429	62.2
H3K56ac Stem (internode) Rep. 2	U071	G11	478	60.4
H3K56ac Vegetative leaf Rep. 2	U072	G12	442	71.4
H3K27me3 Trichomes Rep. 2	U073	H1	436	65.2
H3K27me3 Stem (internode) Rep. 2	U074	H2	448	40.4
H3K27me3 Vegetative leaf Rep. 2	U075	H3	466	51.2

Table S3a. Bowtie2 Alignment mapping statistics of paired end ChIP-seq data alignment to cs10 (**GCF_900626175.2).** Table of the mapping statistics for each ChIP-seq library in this study.

Sample	Total reads	Overall alignment rate	Unmapped Reads	Uniquely mapped reads	Multi-mapping reads
INPUT control Trichomes Rep. 1	25,157,536	90.78%	15.11%	28.31%	56.59%
INPUT control Stem (internode) Rep. 1	21,493,669	91.74%	13.80%	26.23%	59.97%
INPUT control Vegetative leaf Rep. 1	26,893,660	91.47%	14.93%	26.03%	59.04%
H2A.Z Trichomes Rep. 1	12,680,890	82.71%	22.69%	23.60%	53.71%
H2A.Z Stem (internode) Rep. 1	13,028,860	65.50%	39.22%	18.27%	42.50%
H2A.Z Vegetative leaf Rep. 1	18,266,832	86.91%	18.83%	24.37%	56.80%
H3K4me3 Trichomes Rep. 1	28,609,394	89.27%	15.10%	61.42%	23.49%
H3K4me3 Stem (internode) Rep. 1	15,347,697	89.83%	15.57%	51.88%	32.55%
H3K4me3 Vegetative leaf Rep. 1	11,303,989	87.75%	17.95%	36.51%	45.54%
H3K56ac Trichomes Rep. 1	10,440,217	89.65%	16.37%	29.64%	53.99%
H3K56ac Stem (internode) Rep. 1	10,513,921	85.66%	20.23%	29.14%	50.63%
H3K56ac Vegetative leaf Rep. 1	11,569,419	90.44%	15.14%	25.07%	59.79%
H3K27me3 Trichomes Rep. 1	15,697,513	83.17%	22.87%	26.37%	50.76%
H3K27me3 Stem (internode) Rep. 1	14,592,876	83.73%	22.58%	24.95%	52.47%
H3K27me3 Vegetative leaf Rep. 1	10,488,852	79.78%	26.06%	23.18%	50.76%
INPUT control Trichomes Rep. 2	26,716,190	90.85%	14.89%	28.07%	57.04%
INPUT control Stem (internode) Rep. 2	20,994,779	91.71%	14.00%	25.80%	60.19%
INPUT control Vegetative leaf Rep. 2	31,365,486	91.45%	14.51%	26.30%	59.19%
H2A.Z Trichomes Rep. 2	14,816,661	81.30%	23.83%	22.86%	53.31%
H2A.Z Stem (internode) Rep. 2	13,879,251	41.55%	61.47%	11.69%	26.84%
H2A.Z Vegetative leaf Rep. 2	13,114,965	80.28%	25.29%	22.13%	52.58%
H3K4me3 Trichomes Rep. 2	20,846,135	77.82%	25.62%	55.28%	19.11%
H3K4me3 Stem (internode) Rep. 2	11,454,387	89.27%	16.10%	50.85%	33.05%
H3K4me3 Vegetative leaf Rep. 2	13,593,404	90.10%	15.21%	46.31%	38.48%
H3K56ac Trichomes Rep. 2	17,838,025	89.46%	16.49%	30.16%	53.35%
H3K56ac Stem (internode) Rep. 2	12,534,003	79.36%	26.17%	30.05%	43.78%
H3K56ac Vegetative leaf Rep. 2	17,122,515	90.14%	15.68%	26.27%	58.05%
H3K27me3 Trichomes Rep. 2	14,239,146	83.91%	21.83%	25.77%	52.40%
H3K27me3 Stem (internode) Rep. 2	10,228,339	66.16%	38.75%	20.78%	40.48%
H3K27me3 Vegetative leaf Rep. 2	13,828,751	86.62%	19.64%	25.77%	54.60%

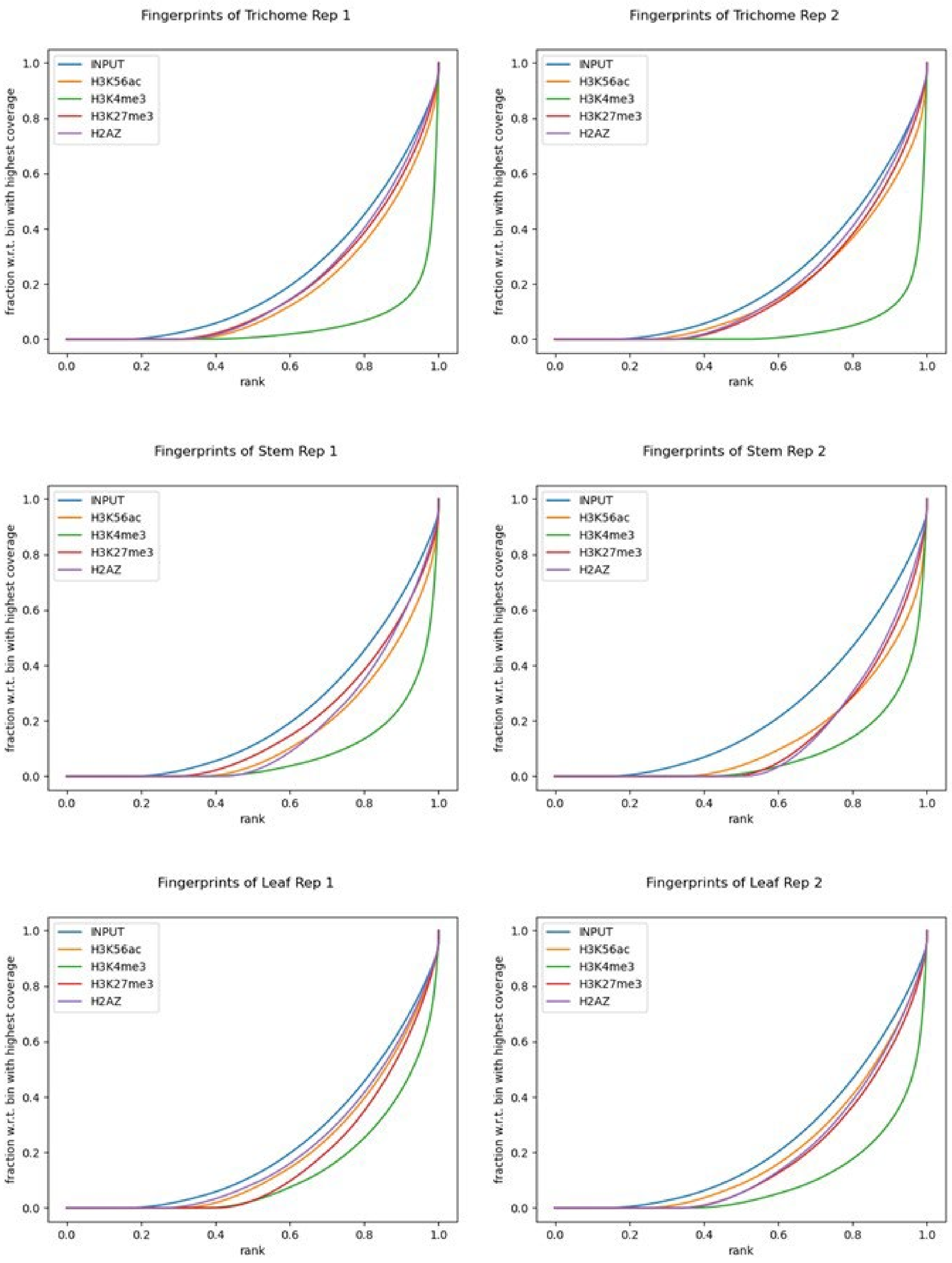

Figure S3. Fingerprint plots of INPUT control and histone marks for each tissue type. Fingerprint plots showing how the sequencing reads of each histone mark distribute across the *C. sativa* genome. Narrow peak data, H3K4me3 in this instance, tend toward acute right handed angles whereby the majority of reads are distributed in a small proportion of the genome and broad histone marks such as H3K56ac, H3K27me3 as well as the histone variant H2A.Z indicate a comparatively linear distribution throughout the genome similar to, yet discriminate from, the distribution of reads observed in the INPUT control

Table S3b. HISAT2 Alignment mapping statistics of single end RNA sequencing data alignment to cs10 (GCF_900626175.2).

Sample	No. of raw reads	No. of mapped reads	%mapped reads
Leaf Rep. 1	23,473,287	21,208,394	90.35
Leaf Rep. 2	24,274,197	21,936,259	90.37
Leaf Rep. 3	23,267,045	20,984,219	90.19
Shoot Rep. 1	27,917,083	25,264,096	90.50
Shoot Rep. 2	19,416,942	17,485,856	90.05
Shoot Rep. 3	25,321,091	22,916,816	90.50
Trichomes Rep. 1	29,914,474	26,944,383	90.07
Trichomes Rep. 2	26,542,737	24,012,723	90.47
Trichomes Rep. 3	25,246,050	22,817,394	90.38

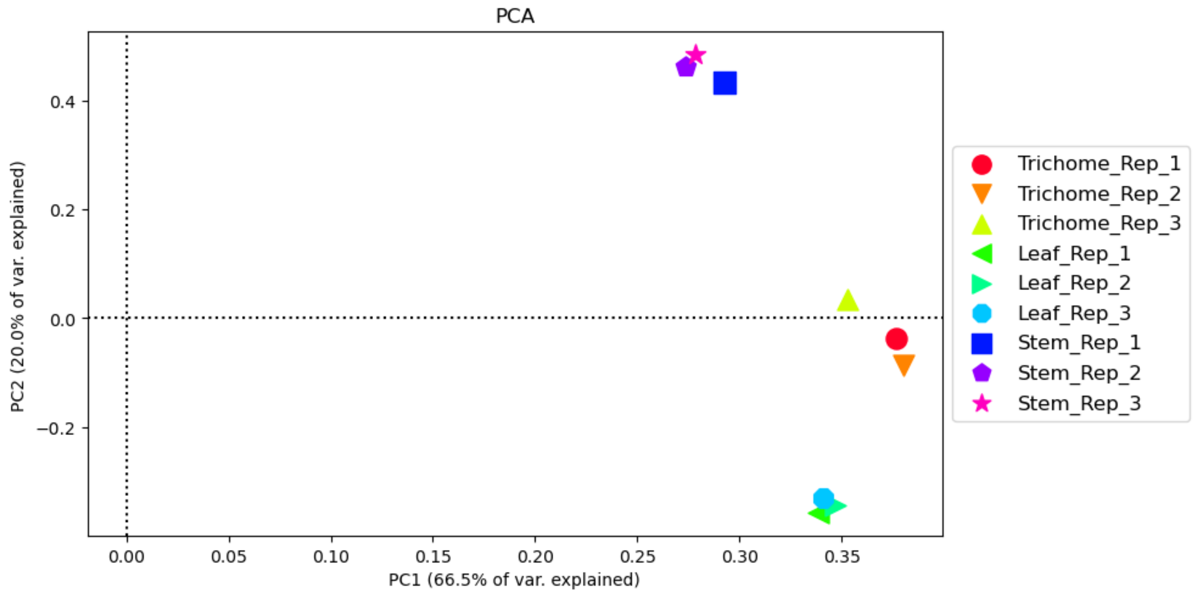

Figure S4. Principal component analysis of RNA sequencing data from glandular trichomes, Stem (internode), and leaf tissues in triplicate. PCA indicates agreement between the biological replicates and distinction between the sample types. 66.5 % of the variance can be resolved by component 1.

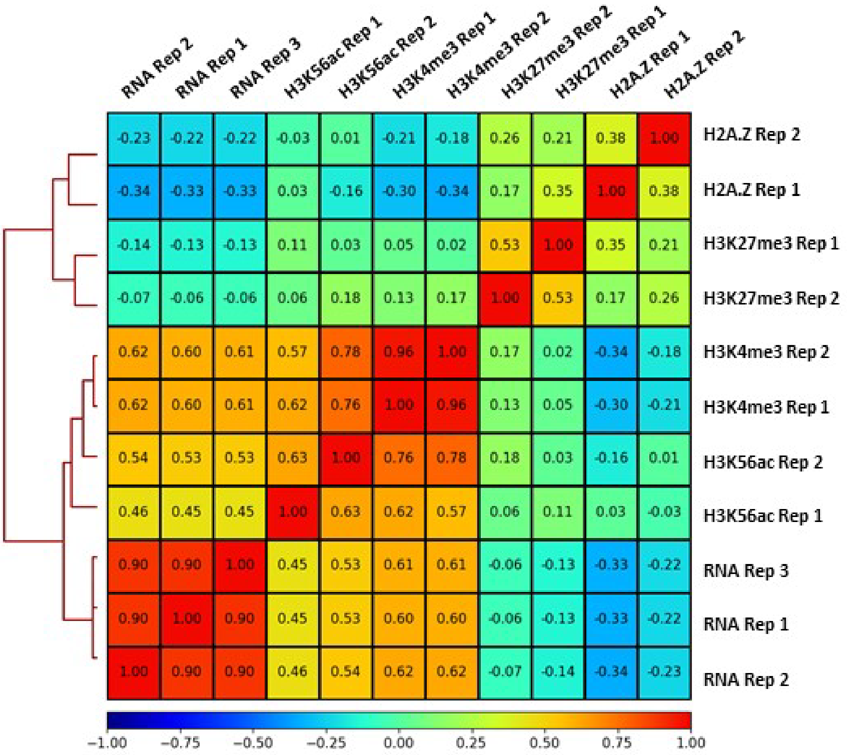

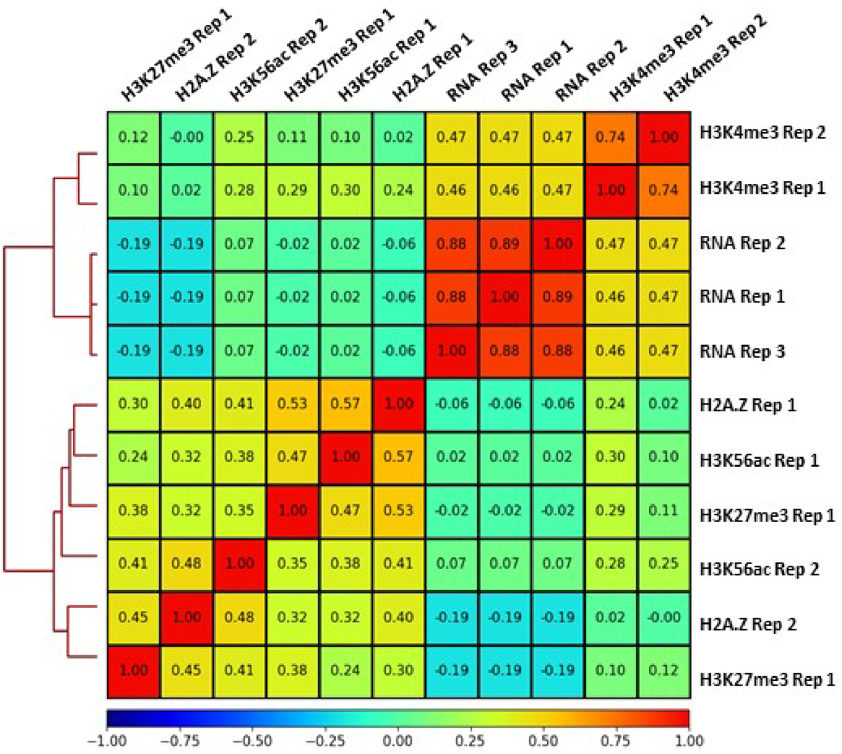

Figure S5. Spearman correlation plots. Data relationships between RNA seq and Histone marks were analysed by spearman correlation and plotted for stem (top) and leaf (bottom). These figures are the stem and leaf compliment of Fig. 1b.

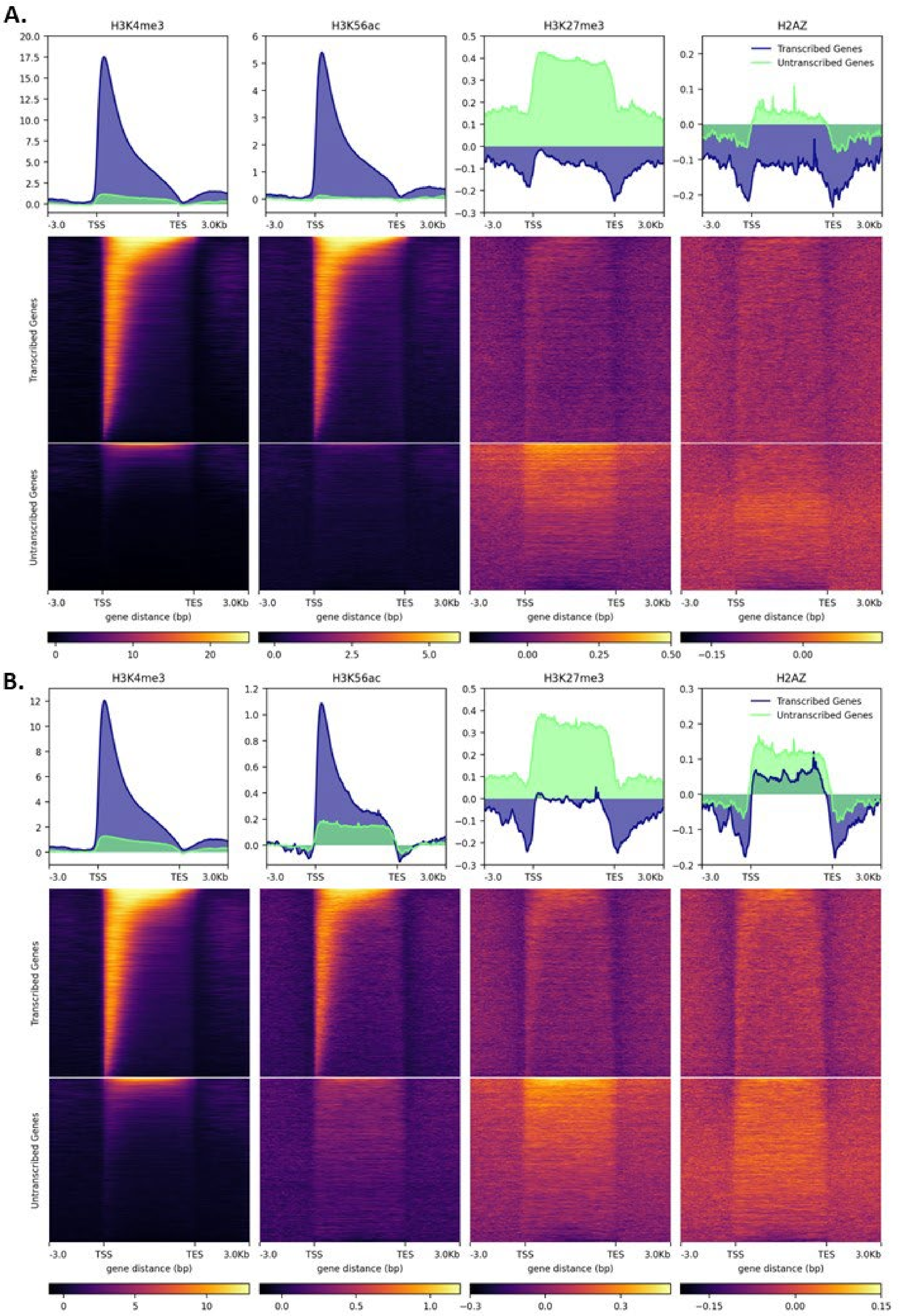

Figure S6. Integrated expression TSS plots of histone marks and variant. Histone mark relationship and distribution in genes transcribed and untranscribed in that respective tissue. Stem (A) and leaf (B). Compliment of trichomes Fig. 1e.

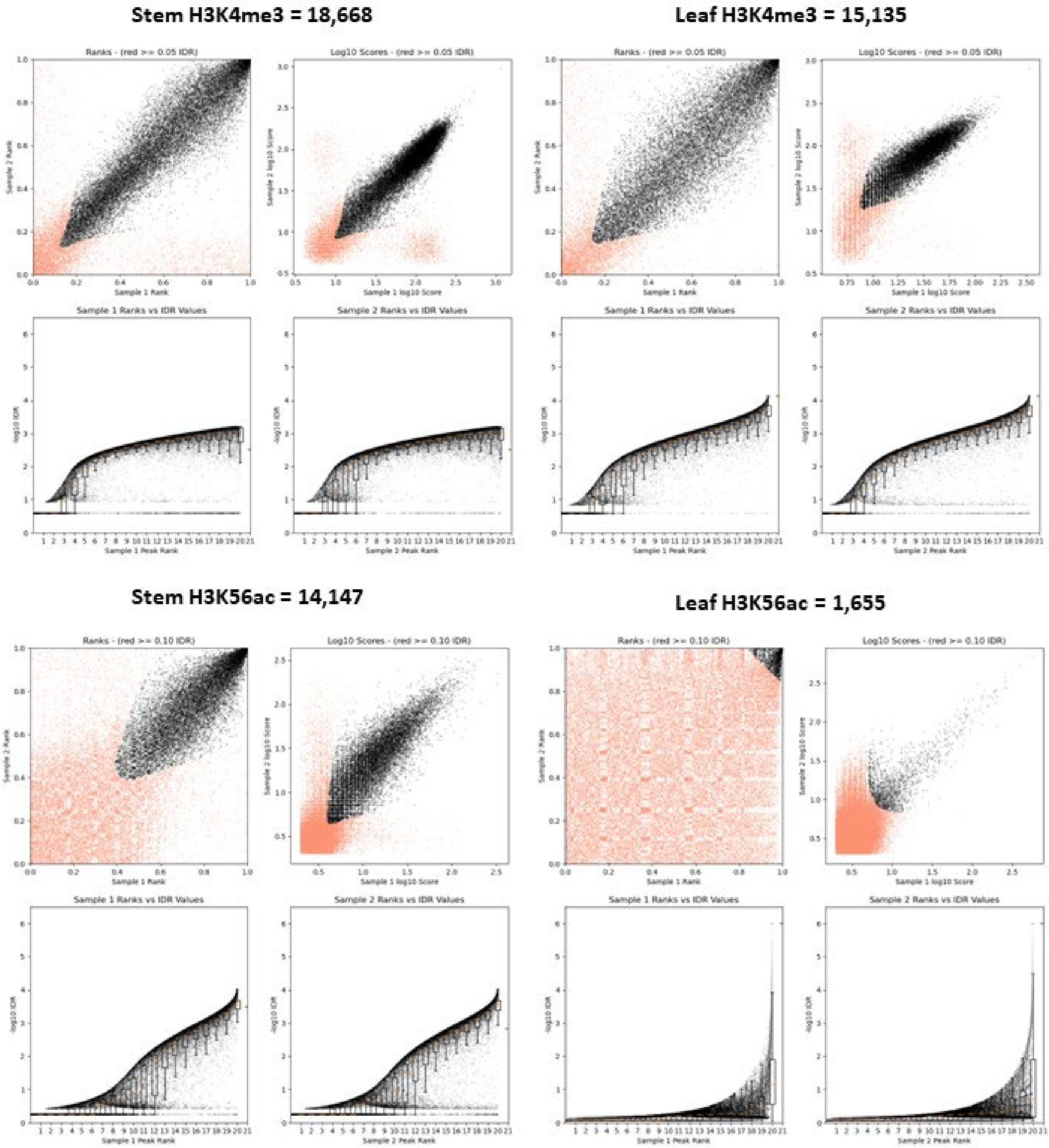

Figure S7. Irreproducible discovery rate analysis of Stem and Leaf. Application of the irreproducible discovery rate analysis method to determine consensus peaks between replicates for both narrow peak H3K4me3 and Mixed peak type H3K56ac replicates for both stem and leaf tissue. Consensus peaks identified are displayed above each panel of four. These are results are the stem and leaf compliment to trichomes found in Fig. 2 a & b.

Number of query intervals (a) H3K4me3 Trichomes: 18774

Number of db intervals (b) H3K56ac Trichomes: 13449

Number of overlaps: 9668

Number of possible intervals (estimated): 333375

phyper(9668 - 1, 18774, 333375 - 18774, 13449, lower.tail=F)

Contingency Table Of Counts

#____

| in -b | not in -b |

in -a | 9668 | 9106 |

not in -a | 3781 | 310820 |

#____

# p-values for fisher's exact test

left	right	two-tail	ratio
1	0	0	87.279

Figure S7b. Fisher exact test results H3K4me3 H3K56ac Trichomes. Null hypothesis is rejected, H3K4me3 and H3K56ac peaks co-occur more than is expected by chance (two-tail p-value < $1x10^{-5}$). With at least 50% reciprocal overlap of peaks.

Number of query intervals: 12601

Number of db intervals: 10028

Number of overlaps: 3169

Number of possible intervals (estimated): 108988

phyper(3169 - 1, 12601, 108988 - 12601, 10028, lower.tail=F)

Contingency Table Of Counts

#____

| in -b | not in -b |

in -a | 3169 | 9432 |

not in -a | 6859 | 89528 |

#____

# p-values for fisher's exact test

left	right	two-tail	ratio
1	0	0	4.385

Figure S7c. Fisher exact test results H3K27me3 H2AZ Trichomes. Null hypothesis is rejected, H3K27me3 and H2AZ peaks co-occur more than is expected by chance (two-tail p-value < $1x10^{-5}$). With at least 50% reciprocal overlap of peaks.

Number of query intervals: 18774

Number of db intervals: 10028

Number of overlaps: 238

Number of possible intervals (estimated): 137167

phyper(238 - 1, 18774, 137167 - 18774, 10028, lower.tail=F)

Contingency Table Of Counts

#____

| in -b | not in -b |

in -a | 238 | 18536 |

not in -a | 9790 | 108603 |

#____

# p-values for fisher's exact test

left	right	two-tail	ratio
0	1	0	0.142

Figure S7d. Fisher exact test results H3K4me3 H3K27me3 Trichomes. Null hypothesis is rejected, H3K4me3 and H3K27me3 peaks co-occur more than is expected by chance (two-tail p-value < $1x10^{-5}$). With at least 50% reciprocal overlap of peaks.

Table S4. Transcribed and untranscribed genes in trichomes that contain histone marks. Transcribed genes are enriched in histone marks H3K4me3 and H3K56ac while untranscribed genes are comparatively depleted. Untranscribed genes show greater enrichment of H3K27me3 and H2A.Z compared to transcribed genes

Genes	H3K4me3 trichome peaks	H3K56ac trichome peaks	H3K27me3 trichome peaks	H2A.Z trichome peaks
Transcribed in Trichomes (17,473)	14,611	10,829	1,395	377
Untranscribed in Trichomes (11,902)	2,009	471	2,366	1,148

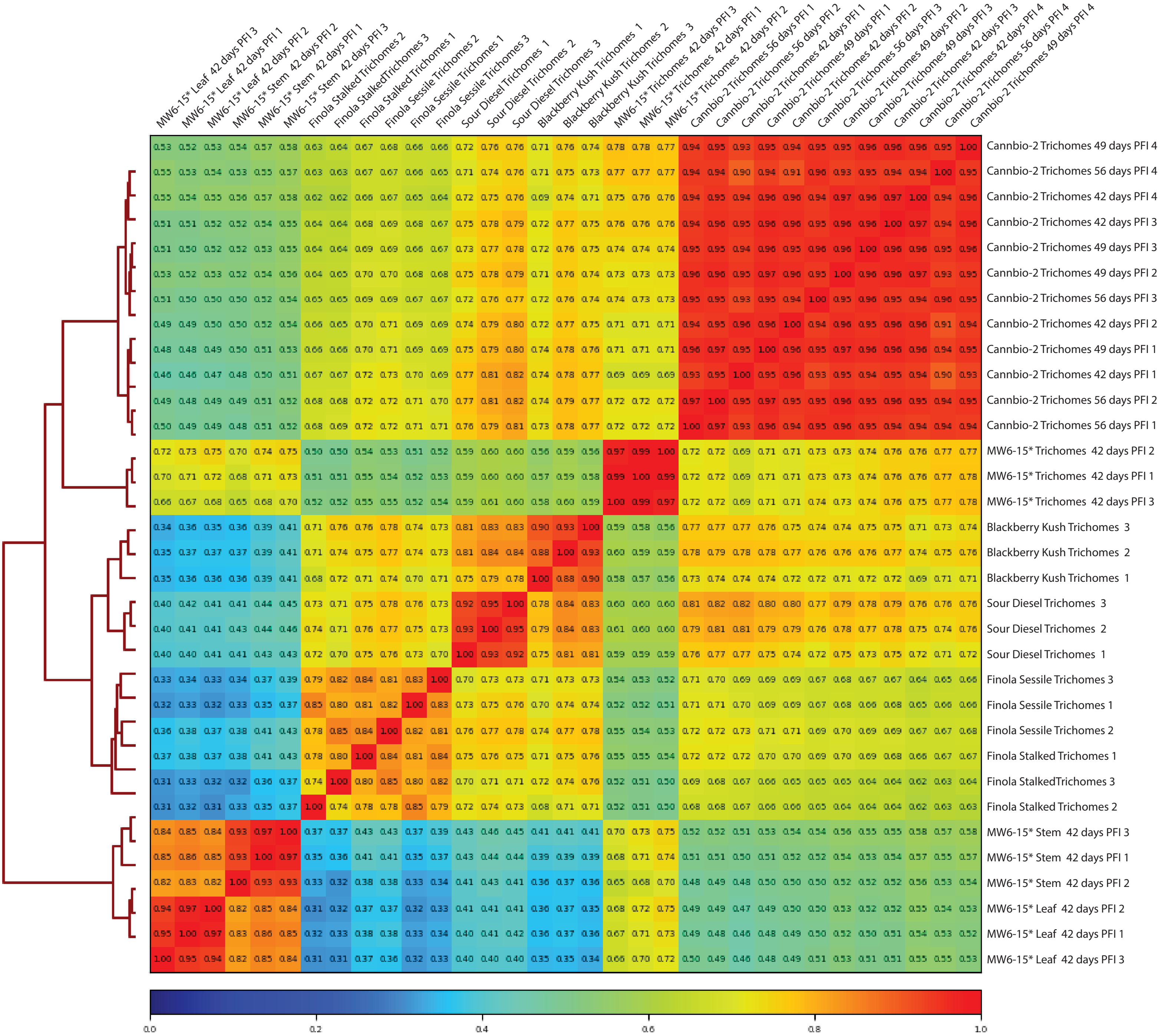

Figure S7e. Spearman correlation plot of publically available Cannabis sativa glandular trichome RNA isolates from 3 independent experiments show tissue specific correlation with the glandualr trichome isolates from this study (MW6-15 Trichomes). Using trichome specific H3K4me3 demarcated genes MW6-15 trichome tissue from our research is shown to strongly correlate with RNA seq datasets from glandular trichome isolates from other studies while leaf and stem RNA datasets form a neat outgroup that do not correlate with any publically available trichome datasets.

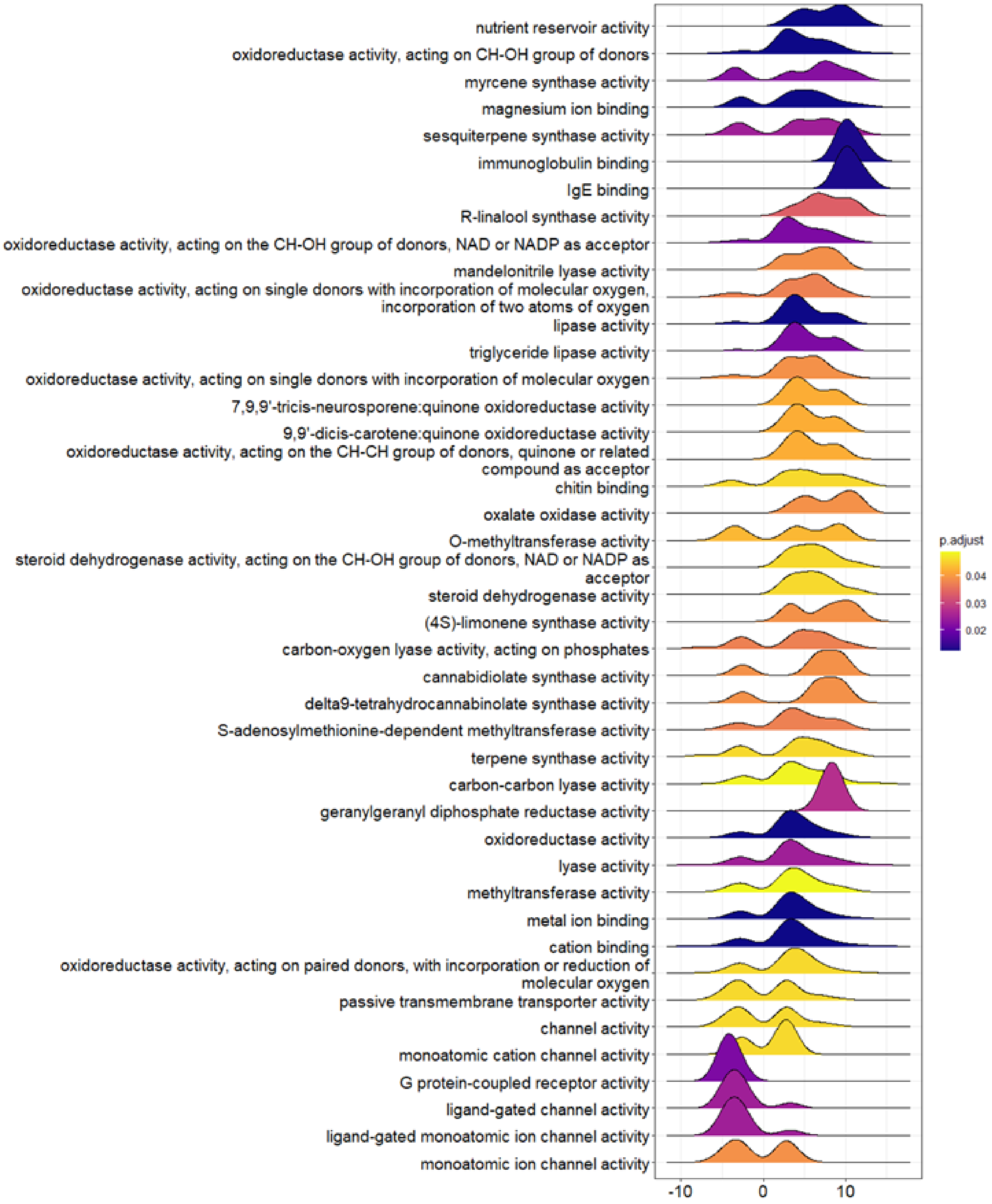

Figure S8a. Ridge plot of glandular trichome enriched gene sets versus leaf. Gene set enrichment analysis of glandular trichomes versus leaf molecular function gene ontologies conveyed by means of a ridge plot. Molecular function ontologies are listed and glandular trichome enriched ontologies are denoted by positive integers on the x-axis.

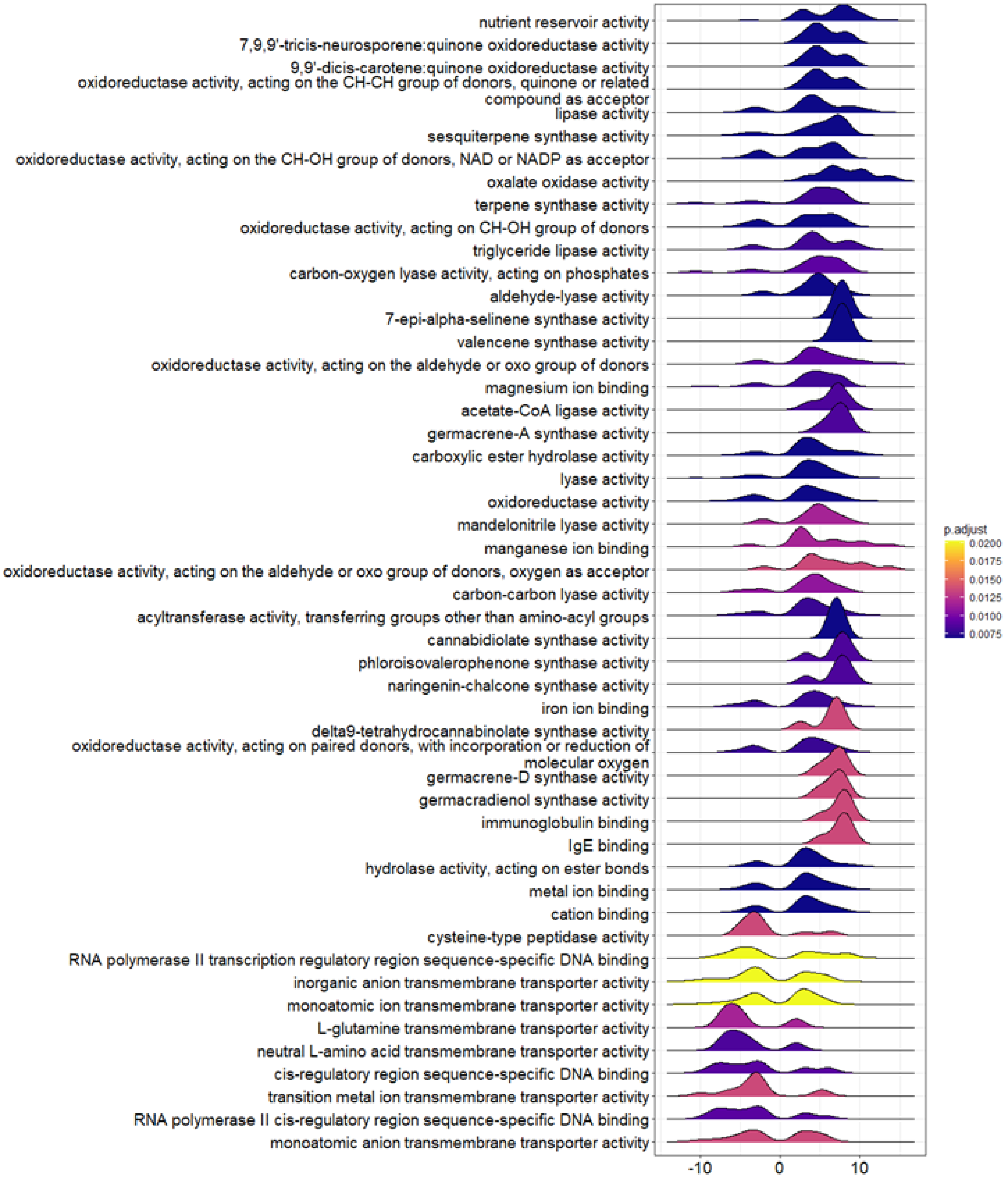

Figure S8b. Ridge plot of glandular trichome enriched gene sets versus stem. Gene set enrichment analysis of glandular trichomes versus stem molecular function gene ontologies conveyed by means of a ridge plot. Molecular function ontologies are listed and glandular trichome enriched ontologies are denoted by positive integers on the x-axis.

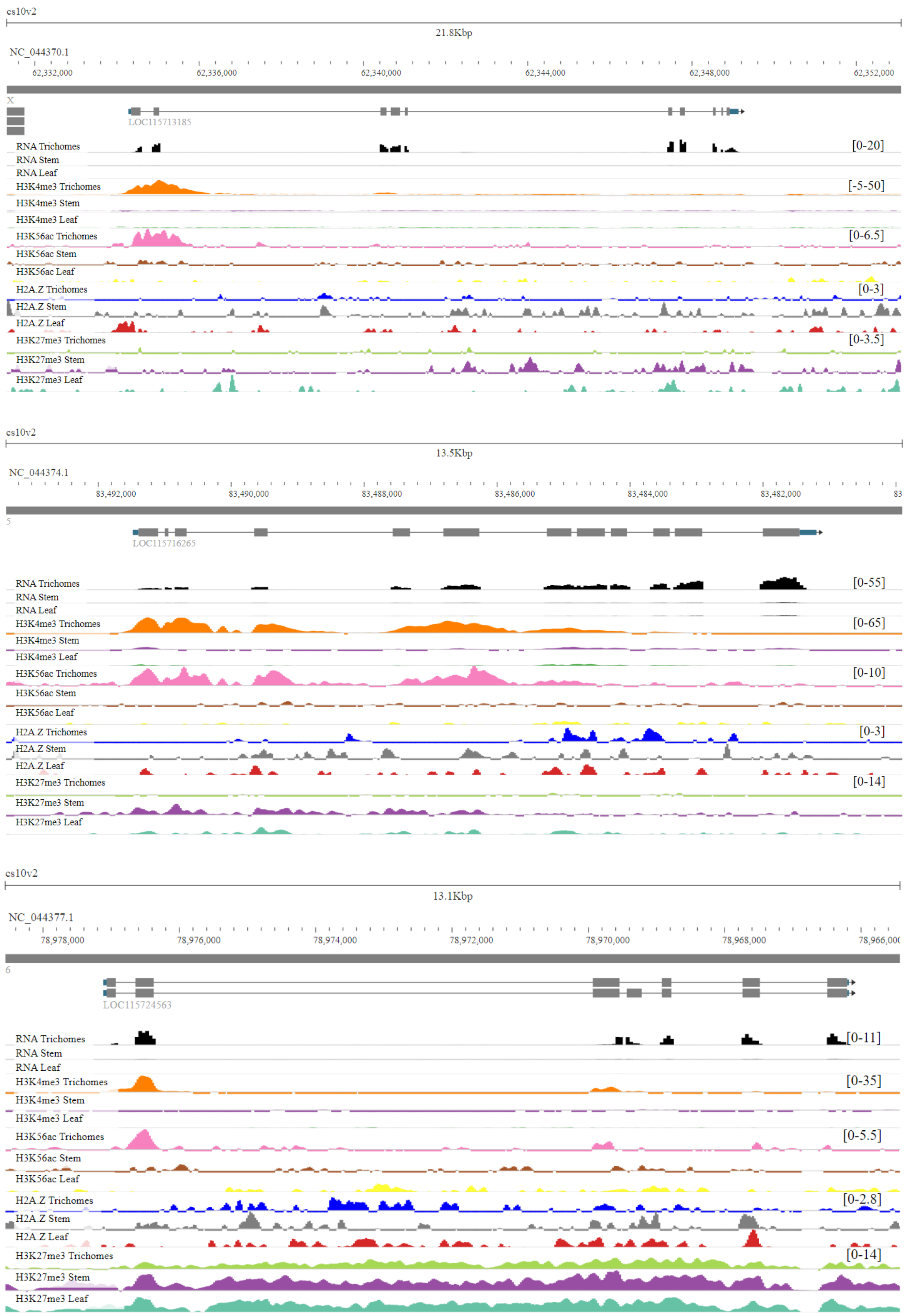

Figure S9a. Three known trichome genes showing tissue-specific expression and chromatin states. LOC115713185 (CBGAS), LOC115716265 (ABC transporter B family member 2), and LOC115724563 (alpha-humulene synthase).

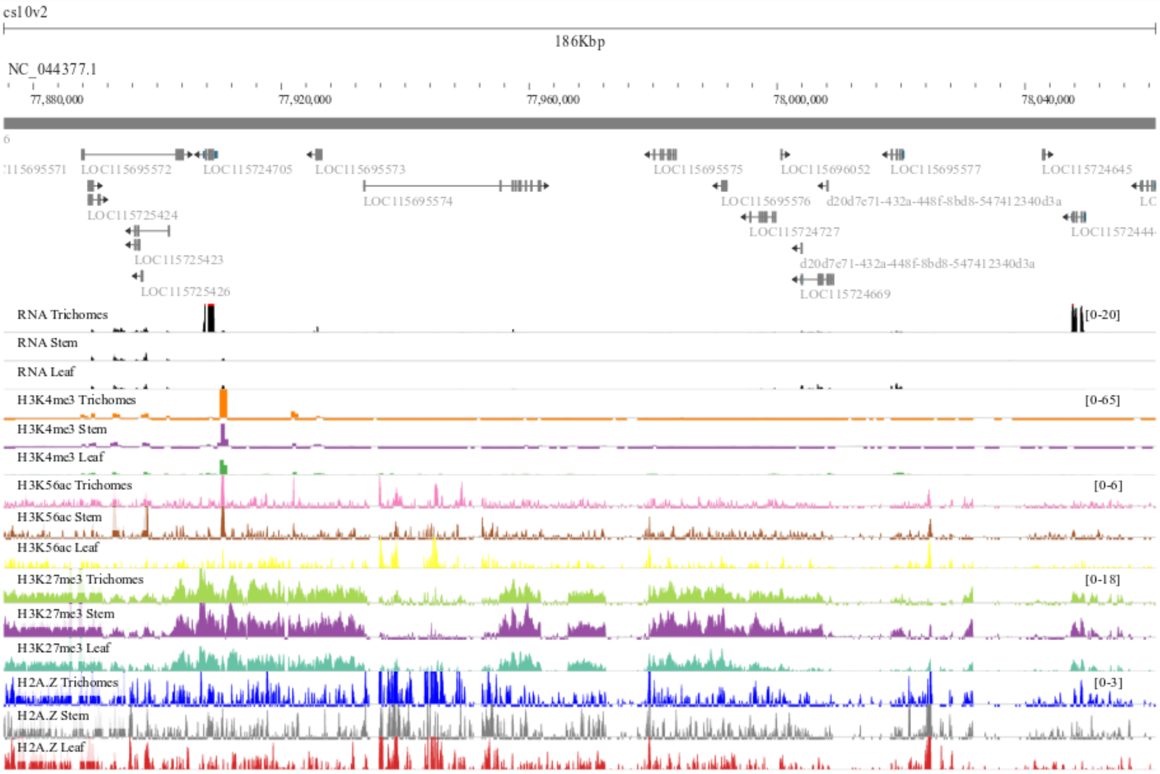

Figure S9b. Biosynthetic gene cluster showing tissue-specific expression. Cluster No. 28 biosynthetic gene cluster that contains genes that play role in plant biotic stress resistance to insects and fungus.
